# Future recovery of baleen whales is imperiled by climate change

**DOI:** 10.1111/gcb.14573

**Published:** 2019-02-26

**Authors:** Vivitskaia J. D. Tulloch, Éva E. Plagányi, Christopher Brown, Anthony J. Richardson, Richard Matear

**Affiliations:** ^1^ ARC Centre of Excellence in Environmental Decisions University of Queensland St Lucia, Brisbane QLD Australia; ^2^ CSIRO Oceans and Atmosphere, Queensland BioSciences Precinct (QBP) St Lucia, Brisbane QLD Australia; ^3^ Australian Rivers Institute, Griffith University Nathan QLD Australia; ^4^ Centre for Applications in Natural Resource Mathematics, School of Mathematics and Physics The University of Queensland St Lucia QLD Australia; ^5^ CSIRO Oceans and Atmosphere Hobart Tasmania

**Keywords:** Antarctic, ecosystem model, fisheries, global warming, migration, Multispecies model, predator–prey interactions, whaling

## Abstract

Historical harvesting pushed many whale species to the brink of extinction. Although most Southern Hemisphere populations are slowly recovering, the influence of future climate change on their recovery remains unknown. We investigate the impacts of two anthropogenic pressures—historical commercial whaling and future climate change—on populations of baleen whales (blue, fin, humpback, Antarctic minke, southern right) and their prey (krill and copepods) in the Southern Ocean. We use a climate–biological coupled “Model of Intermediate Complexity for Ecosystem Assessments” (MICE) that links krill and whale population dynamics with climate change drivers, including changes in ocean temperature, primary productivity and sea ice. Models predict negative future impacts of climate change on krill and all whale species, although the magnitude of impacts on whales differs among populations. Despite initial recovery from historical whaling, models predict concerning declines under climate change, even local extinctions by 2100, for Pacific populations of blue, fin and southern right whales, and Atlantic/Indian fin and humpback whales. Predicted declines were a consequence of reduced prey (copepods/krill) from warming and increasing interspecific competition between whale species. We model whale population recovery under an alternative scenario whereby whales adapt their migratory patterns to accommodate changing sea ice in the Antarctic and a shifting prey base. Plasticity in range size and migration was predicted to improve recovery for ice‐associated blue and minke whales. Our study highlights the need for ongoing protection to help depleted whale populations recover, as well as local management to ensure the krill prey base remains viable, but this may have limited success without immediate action to reduce emissions.

## Graphical abstract

We predict impacts of historical commercial whaling (black bars, top), and future climate change, including changing sea‐surface temperature (shown in red, top), primary productivity and sea‐ice extent, on populations of baleen whales and their prey (krill and copepods) across the Southern Hemisphere. Despite initial recovery from commercial harvesting, models predict concerning declines for whale populations due to climate change by 2,100 for populations of blue (blue bars, bottom), fin (green, bottom), humpback (purple, bottom) and Southern right whales (orange, bottom) as a consequence of reduced krill prey (grey line) from warming.

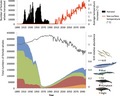

## 1 INTRODUCTION

**Figure 1:**
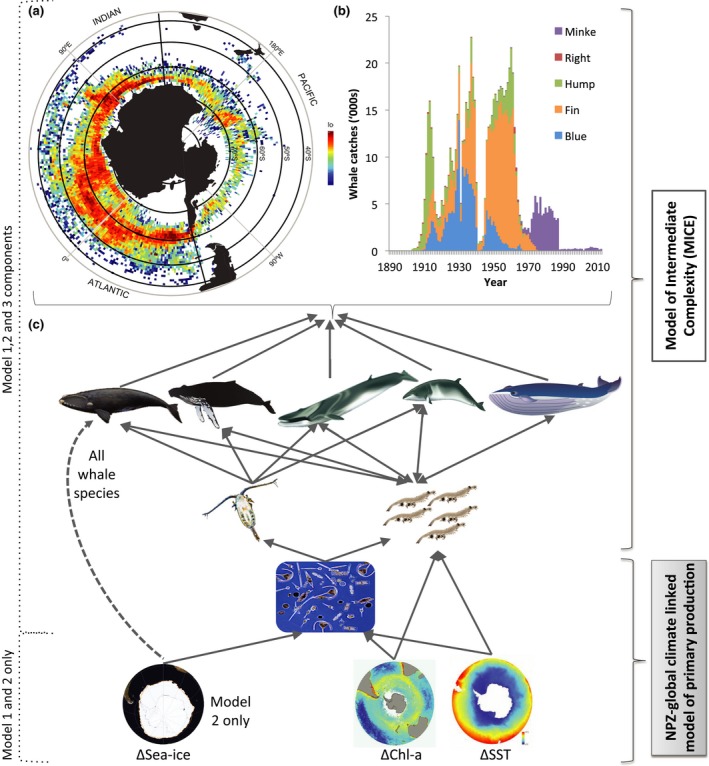
Historical whale harvests shown by (a) heat map, where black circumpolar bands identify the four latitude bands used in the model, and thick black lines at 60°W and 130°E identify breaks between the two oceanic regions modelled, and (b) stacked column graph of total harvest over time between 1890 and 2015; (c) schematic of direct interactions between physical climate drivers (bottom from left—changes in sea ice, chlorophyll, sea‐surface temperature) and biological features of models (phytoplankton, copepods, krill and whales) detailing the relationships between the primary model that included environmental forcing from temperature and phytoplankton (Model 1) and alternative scenarios that added links between changing sea ice and future whale distribution (Model 2) and where all climate drivers were excluded (Model 3). Arrows identify the direction of the driver and/or interaction; whales depicted from left to right are southern right, humpback, fin, minke and blue [Colour figure can be viewed at http://wileyonlinelibrary.com]

Historical commercial whaling in the Southern Hemisphere, particularly in the Southern Ocean, pushed most whale species to the brink of extinction (Figure 1a, b) (Clapham & Baker, 2002; May, Beddington, Clark, Holt, & Laws, 1979; Tulloch, Plagányi, Matear, Brown, & Richardson, 2017). After the cessation by the 1980s of most harvesting in the Southern Hemisphere due to a moratorium on whaling imposed by the International Whaling Commission (IWC), depleted populations began to slowly recover. Although the protection measures avoided extinction of baleen whales, new pressures from human‐induced climate change are now affecting the marine environment and potentially the species within (Constable et al., 2014). It is not yet known how future climate change may facilitate or hinder the recovery of whales, particularly those that forage heavily on krill in rapidly warming regions of the Southern Ocean (Simmonds & Isaac, 2007; Smetacek & Nicol, 2005).

The climate at some polar regions is changing faster than any other region in the world (Meredith & King, 2005). Changes in the Southern polar region are not uniform, varying regionally and temporally (Massom & Stammerjohn, 2010; Matear, O'Kane, Risbey, & Chamberlain, 2015), with increases in Antarctic sea‐ice extent and cooling in regions such as the Ross Sea (Turner, Hosking, Bracegirdle, Marshall, & Phillips, 2015), compared to significant sea‐ice losses due to warming in the west Antarctic Peninsula (Stammerjohn, Martinson, Smith, & Yuan, 2008; Stammerjohn, Massom, Rind, & Martinson, 2012). Warming has already impacted low trophic forage species such as krill (Atkinson, Siegel, Pakhomov, & Rothery, 2004; Kawaguchi et al., 2011), which has undergone a 30% decline in density in some areas such as the Scotia Sea since the 1980s (Atkinson et al., 2004), and future warming and changes in primary productivity are expected to accelerate. Changes in polar species phenology, survival and southward range shifts driven by warming oceans are predicted (Clarke et al., 2007; Smith et al., 1999). Shifts in food webs from the base to top predators in the West Antarctic Peninsula have already been observed (Ducklow et al., 2007; Schofield et al., 2010).

Baleen whales in the Southern Hemisphere are particularly dependent on stable environmental conditions and sustenance from polar waters, travelling long distances from nursery grounds in lower‐latitude warmer waters to high‐latitude feeding grounds in the Antarctic where their primary prey species krill (*Euphausia superba*) and other zooplankton prey are found in high numbers. Their slow population growth rates, tight synchrony between life history and water temperatures, and dependency on lower trophic level prey, such as krill linked directly to primary productivity, may make baleen whales likely to be particularly sensitive to future climate change (Leaper et al., 2006). However, the exact mechanisms of change are still unclear as little is known of the dynamics among interacting species in Antarctic ecosystems. Recent research shows that global climate indices influence southern right whale breeding success by determining variation in food (krill) availability (Seyboth et al., 2016). Changes in predator populations in response to climate‐induced changes in their environment and/or prey have been observed for other species in the Southern Hemisphere. Local declines in ice‐dependent Adelie (*Pygoscelis adeliae)*penguins versus increases in ice‐intolerant gentoo (*Pygoscelis papua*) and chinstrap (*Pygoscelis antarctica*) penguins in the West Antarctic Peninsula have been attributed by some to a climate shift, with increasing maritime influences from the north affecting ice availability and snow accumulation (Ducklow et al., 2007; Fraser, & Patterson, 1997; Fraser & Trivelpiece,^1996^; Smith et al., 1999). Alternative hypotheses, however, purport availability of and competition for krill, the dominant prey for nearly all vertebrates in the Antarctic, is more likely driving historical and present changes in predator numbers throughout the region (Trivelpiece et al., 2011). Euphausiids are known to be the major food source of baleen whales in the Southern Ocean (Laws, 1985), but we have little in situ knowledge of interspecific and intraspecific competitions among those whales for food (Kawamura, 1994). What is clear from the examples given for these other krill‐dependent species is that spatial and/or temporal mismatches between species life history or phenology and food or habitat availability play a large role in structuring polar ecosystems and species populations. This match–mismatch paradigm (Anderson, Gurarie, Bracis, Burke, & Laidre, 2013; Cushing, 1974) may be key to understanding and predicting effects of climate change on the survival, growth and reproduction of higher tropic levels of the marine food web.

For baleen whales, a number of hypotheses have been posited with regards to how their demography and phenology may respond to climate change. Warming in lower latitudes may cause contractions in migration ranges (similar to the phenotypic plasticity observed in some migratory bird species, e.g. Pulido and Berthold (2010), Klaassen et al. (2014)), which may directly increase juvenile survival by potentially altering the shape of the fitness surface (Reed, Schindler, & Waples, 2011). These benefits, however, would be traded‐off if warming and sea‐ice reduction caused migratory whales to range farther to find prey, increasing energy expenditure and decreasing fitness (Moore & Huntington, 2008). As the extent and exact nature of the reliance of ice‐associated species such as blue and minke whales on sea‐ice–mediated ecosystems in the Antarctic remains unclear (Laidre et al., 2008), it is not known how changing sea‐ice extent may affect these species in the Southern Hemisphere. In the Northern Hemisphere, there is some evidence to suggest flexible foraging species such as gray whales have begun to adapt to warming and the reduction in winter sea ice by remaining in Arctic areas over winter (Moore, Wynne, Kinney, & Grebmeier, 2007). In contrast, warming and associated decreased prey availability are expected to have negative consequences on endangered North Atlantic right whales given recent indications of northward range shifts in their prey (Meyer‐Gutbrod & Greene, 2018). Although some populations of ice‐associated species such as bowhead whales have increased despite sea‐ice loss (George, Zeh, Suydam, & Clark, 2004), suggesting sea‐ice reduction has not hindered recruitment and may be expanding foraging opportunities (Moore & Huntington, 2008), other research suggests increased thermal stress and habitat loss associated with warming may have serious impacts on bowhead whale populations (Chambault et al., 2018). With many of the large whales still at depleted levels in the Southern Hemisphere due to massive commercial catches throughout the 20th century (see Tulloch et al., 2017), an improved understanding of how they may respond to changes in their environment and prey from additional anthropogenic stressors is vitally important from a conservation perspective.

There is a paucity of examples of ecosystem models linking marine environmental change and associated changes in primary productivity to krill and their predators in the Southern Hemisphere (Klein, Hill, Hinke, Phillips, & Watters, 2018; Tulloch et al., 2017). Earlier models have investigated the recovery of baleen whales from harvesting (Mori & Butterworth, 2006; Tulloch et al., 2017), and explored the role trends in krill populations may have in predator recovery (Klein et al., 2018; Plagányi & Butterworth, 2012; Watters, Hill, Hinke, Matthews, & Reid, 2013). An integrated assessment of whale recovery under climate change is missing that links future recovery of individual whale species to multiple climate drivers and changing food availability (krill and copepods) across the Southern Hemisphere.

Here, we assess impacts of two anthropogenic pressures on baleen whales—historical whaling and future climate change—across the Southern Hemisphere, using a multispecies Model of Intermediate Complexity for Ecosystem assessment (MICE) (Plagányi et al., 2014). We build on an earlier MICE of whale recovery that included primary productivity (phytoplankton), krill, copepods (the most abundant small zooplankton) and five baleen whale predators: the southern right whale *Eubalaena australis*, the humpback whale *Megaptera novaeangliae,* the Antarctic minke whale *Balaenoptera bonaerensis*, the fin whale *Balaenoptera physalus* and the Antarctic blue whale *Balaenoptera musculus* (Figure 1c, Tulloch et al., 2017). MICE are well suited for developing predictions of large‐scale system dynamics requiring an understanding of interactions between species and processes because these models restrict focus to the key ecosystem components required to answer the question and can account for key uncertainties through parameter estimation based on fitting to data and sensitivity analyses.

The ecosystem model links to an existing Nutrient–Phytoplankton–Zooplankton–Detritus model (NPZD) forced by a General Circulation Model that includes ocean and atmosphere dynamics (Law et al., 2017; Ziehn, Lenton, Law, Matear, & Chamberlain, 2017). We extend earlier models (Tulloch et al., 2017) to include links from projected sea‐surface temperature (SST) and chlorophyll to the whale prey base and whale species dynamics, to evaluate potential impacts of multiple climate drivers on key baleen whale species and their krill prey across the Southern Hemisphere. Our model further builds on previous research (Tulloch et al., 2017) by including two‐way interactions to explore how competition for limited prey may differentially affect whale populations in the future. We then consider how future changes in sea‐ice extent given warming might affect whale populations if species are able to shift their range to access more favourable environmental conditions.

## 2 MATERIALS AND METHODS

We used a model of intermediate complexity (MICE) for five baleen whale species (blue, fin, humpback, southern right and Antarctic minke), and their krill and copepod prey (Tulloch et al., 2017). We extend the model to include two‐way predator–prey interactions and allow for three alternative versions that include or exclude links with climate change (Figure 1). In the primary model (Model 1), we coupled krill dynamics to future changes in SST and chlorophyll, with indirect links between climate and whales through changing prey availability and whale breeding success (Figure 1, Table 1). In Model 2, we included all links from Model 1 among climate, prey and whales, but added links between future changes in sea‐ice extent to hypothesized changes in whale distribution (Figure 1, Table 1). Finally, we ran a comparative scenario (Model 3) where climate drivers were decoupled from species dynamics, and changes in whale and krill numbers were driven by predation and competition between whales alone (Figure 1, Table 1). All models were fitted to an index of abundance from available surveys for krill and the five whale species (Tulloch et al., 2017) using AD Model Builder (Fournier et al., 2012). The models first simulate historical whale trajectories from 1890 to 2013 for two regions (Pacific and Atlantic/Indian) and two seasons (feeding and breeding, Figure S1), driven largely by the historical commercial whaling records from the IWC (Figure 1a, b). The model then predicts krill and whale numbers to the end of the 21st century driven by projections from the NPZD model. The NPZD model future predictions were coupled to climate drivers under Representative Concentration Pathways (RCP) 8.5 (Meinshausen et al., 2011) adopted by the Intergovernmental Panel on Climate Change (IPCC) Fifth Assessment Report (AR5) (IPCC, [Link]) (Figure 1c). We use this single scenario because climate estimates are currently tracking the highest RCP pathway 8.5 (Sanford, Frumhoff, Luers, & Gulledge, 2014), but the projected changes here might be considered an upper bound if emissions were dramatically reduced.

**Table 1:** Direct and indirect interactions among climate drivers, krill and whales included in the MICE models (see also Figure 1). Shaded boxes indicate that the effect is included in the model design

	Krill			Whales			
Driver	Climate‐driven changes in primary productivity	Sea‐surface temperature change	Chlorophyll (Chl a) change	Climate‐driven changes in primary productivity	Sea‐surface temperature change	Chlorophyll (Chl a) change	Sea‐ice thickness
Direct/indirect interaction with climate drivers	Indirect	Direct	Direct	Indirect	Indirect	Indirect	Direct
Mechanism of interaction in model	Food availability (modelled through recruitment term)	Growth parameter	Growth parameter	Food availability (through predator–prey interaction term)	Prey availability (through predation)	Prey availability (through predation)	Range restriction (adjusts distribution latitudinally)
Key references to substantiate interaction	Clarke (1988), Atkinson et al. (2006)	Constable et al., (2014), Hill et al., (2013), Murphy et al., (2007), Wiedenmann et al., (2008)	Atkinson et al. (2006)	Laws (1985), Kawamura (1994), Reilly et al. (2004)	Laws (1985), Kawamura (1994), Reilly et al. (2004)	Laws (1985), Kawamura (1994), Reilly et al. (2004)	Moore et al. (2007), Simmonds and Eliott (2009), Moore and Huntington (2008)
Model 1							
Model 2							
Model 3							

The MICE includes four spatial zones, delimited by splitting the Southern Hemisphere into two oceanic Areas corresponding to the Atlantic/Indian Oceans (and corresponding Southern Ocean region) (130°E–60°W) and the Pacific Ocean and Southern Ocean region (60°W–130°E). These zones followed standards used in previous Southern Ocean ecosystem models (Branch, Matsuoka, & Miyashita, 2004; Mori & Butterworth, 2006), which follow whale stock management boundaries established by the IWC. Each region was then split into two seasons—winter tropics (0–40°S) and summer polar (40–80°S, Figure S1). The southern “polar” area extending to Antarctica corresponds to the austral summer (November–April) where whales are present in their polar feeding grounds and the northern winter “tropics” area to the equator covers the annual whale migration north to warmer waters (May–October). We further separated the summer polar region into four 10° Latitude bands (*L*) from 40°S to 80°S.

We derived historical (1900–2000) and future (2000–2100) model projections for mean standing phytoplankton biomass (mmol.m^−3^) for the top 50 m of the ocean, mean SST to a depth of 20 m (Hill, Phillips, & Atkinson, 2013), mean sea‐ice mass (kg.m^−2^) and relative concentration proportion (0–1) from the Australia Community Climate and Earths System Simulator (ACCESS (Law et al., 2017, Ziehn et al., 2017)), aggregated by each Latitude band and Area, for the summer whale feeding and krill growth period (November–April). We calculated the maximum phytoplankton biomass for each Area, using this to scale phytoplankton biomass in each year as relative to this maximum ($\rho_{L,y}^{rel,A}$). Phytoplankton was converted into annual average chlorophyll (mmol.m^−3^) per Latitude band assuming a Nitrogen‐to‐Carbon (16:106) and a Carbon‐to‐Chlorophyll (50:1) conversion ratio (Redfield, 1934). We divided ice mass by concentration and converted the volume to obtain annual mean ice thickness (m) used as a relative ice‐thickness multiplier to evaluate the effect of changing sea ice on whales.

The spatial disaggregation of climate and prey enabled evaluation of the match–mismatch hypothesis (Anderson et al., 2013; Cushing, 1974), namely, how climate variability might differentially affect krill biomass and hence impact whales.

### 2.1 Prey dynamics (krill and copepods)

For krill dynamics we expand on an existing age‐structured population model by Kinzey, Watters, and Reiss (2015), given by:

(1a) $$N_{L,y+1,0}^{\text{krill,}A}=R\left(B_{L,y}^{sp,A}\right)$$

(1b) $$N_{L,y+1,a+1}^{krill,A}=N_{L,y,a}^{krill,A}e^{-M}\cdot \theta_{L,y}^{A}\;0\leq a\leq z-2$$

(1c) $$N_{L,y+1,z}^{krill,A}=\left(N_{L,y,z}^{krill,A}e^{-M}+N_{L,y,z-1}^{krill,A}e^{-M}\right)\cdot \theta_{L,y}^{A}$$

where $N_{L,y,a}^{krill,A}$ is the number of krill in area *A* and Latitude *L*, of age *a* at the start of Model year *y*, $R(B_{L,y}^{sp,A})$ is the krill recruitment as a function of spawner biomass (note recruits are defined as 1 year old) in area *A* and Latitude *L*per year *y*, *M* is the (time‐invariant) input natural mortality rate for krill (input, see Table S2), *z*is the largest age considered and $\theta_{L,y}^{A}$ is the standardized consumption of krill by whales in area *A*and Latitude *L*in year *y*(Tables S3).

We built upon previous research (Tulloch et al., 2017) by including a consumption term $\theta_{L,y}^{A}$ to examine two‐way interactions between the whales and their prey over time. A large body of evidence exists substantiating the feeding habits of baleen whales on krill and copepods, and resulting energy transfer through the food web (Laws, 1985; Reilly et al., 2004). We estimated average annual per capita consumption *C*of krill (tonnes) per individual whale per year from the literature, using estimates from Ratnarajah et al. (2016) for blue, fin and humpback whales (Table S3). For minke whales we calculated consumption by scaling up estimates of consumption derived for individual minke whales in the Ross Sea, Areas III and VI (Tamura & Konishi, 2006, 2009) and Areas IV and V (Tamura, Ichii, & Fujise, 1997). For southern right whales, some estimates of total krill consumed in the Southern Hemisphere have been derived (2,253–2,600 × 10^3^tons/year) (Perrin & Wursig, 2009), from which we calculated approximate consumption values per area, scaled to total krill consumed by an individual whale by dividing by the proportion of Southern right whales in each area and their current population estimate.

We scaled *C* by dividing by the krill carrying capacity, or starting biomass of krill $B_{L,0}^{sp,A}$ in each Latitude and Area, to find the average proportion of krill consumed at equilibrium $Q_{L}^{ave,A}$ (assumed to be when whales and krill are at carrying capacity). Starting krill biomass for the entire region was set at 379 Mt (Atkinson, Siegel, Pakhomov, Jessopp, & Loeb, 2009), and spatially disaggregated to derive pre‐exploitation spawning biomass of krill per Latitude and Area based on maps of observed circumpolar distribution of Antarctic krill (Hill et al., 2013). We multiplied the consumption *C* value by the number of whales of species *j*in feeding area *A* in year *y,* $N_{y}^{j,A}$ (described below), and relative proportional summer spatial distribution in each Latitude and Area for whale species *j* ($\nu_{L}^{j,A}$, see Figure S2) to calculate the proportion of krill eaten by each whale species $Q_{L,y}^{j,A}$, updated annually, as follows:

(2) $$Q_{L,y}^{j,A}=\left(C_{L}^{j,A,krill}\;/B_{L,0}^{sp,A}\right)\cdot N_{y}^{j,A}\cdot v_{L,y}^{j,A}$$

We assumed whales feed on the larger‐sized krill (age 4 and older) as this was the best representation of likely ages or sizes eaten. Finally, we use this value to generate the consumption multiplier term $\theta_{L,y}^{A}$ using the following equation, which calculates the difference between standardized krill consumption at equilibrium ($Q_{L}^{ave,A}$) and that at each time step (and hence is used to determine whether krill consumption is above or below the equilibrium level):

(3) $$\theta_{L,y}^{A}=1-\left(\sum_{j}Q_{L,y}^{j,A}-Q_{L}^{ave,A}\right)$$

The whale distribution parameter $\nu_{L,y}^{j,A}$, or relative proportion of each whale population *j* distributed (on average) in each of the 10° Latitude bands in each Area during the summer feeding months, was first estimated and fixed based on the historical catch distribution (Allison, 2013; see also Tulloch et al., 2017). We also collated distribution information from the literature for feeding areas of all whale species and prey preferences (krill vs. other prey species such as copepods, derived in Tulloch et al., 2017). We validated and adjusted the mean historical whale distribution per Latitude based on their upper and lower latitudinal feeding limits, and latitudinal midpoint for feeding, according to the literature (Figure S2).

The krill spawning biomass $B_{L,y}^{sp,A}$for all three models assumes independence of Latitude bands in terms of recruitment, using a knife‐edge maturity‐at‐age function, with 100% maturity at 49 mm (age 4) (Siegel & Loeb, 1994), as follows:

(4) $$B_{L,y}^{sp,A}=\sum_{a=4}^{z}w_{L,y,a}^{A}N_{L,y,a}^{krill,A}$$

where the relationship between carapace length *l* (mm) and krill whole wet mass *w* (grams) of animals of age *a* in latitude *L* of area *A* in year *y* was based on the following power relationship refined using Equation 3 from Hewitt, Watkins et al. (2004):

(5) $$w_{L,y,a}^{A}=2.236\cdot 10^{-6}\cdot {\left(\ell_{L,y,a}^{A}\right)}^{3.314}$$

To convert krill length into age, we used a von Bertalanffy growth equation to relate carapace length *l*(mm) to age in years (*t*), based on Siegel (1987), as follows:

(6) $$\ell_{L,y,a}^{A}=\ell_{\infty }\left[1-e^{-\kappa \left(t-t_{0}\right)}\right]\cdot GR_{L,y}^{A}$$

where $\ell_{\infty }$ was the maximum length of krill (mm), κ and $t_{0}$ were krill growth rate parameters from Hill et al. (2013) (parameter values input, see Table S2). This yields the average length of an animal of age *a*, but we scale the value upwards or downwards based on an annual growth rate $GR_{L,y}^{A}$ derived from NPZD climate outputs for each region relative to the start year growth rate, described below.

To explore how krill in our model could respond to changes in the environment and food availability, we first collated a summary of known direct responses of krill to changes in physical parameters (changing SST; changes in sea‐ice extent, duration and thickness; productivity‐driven variability; increasing CO_2_ and ocean acidification; and UV/irradiance), based on experimental and observational literature (Table S5). Experimental approaches have shown that environmental variability affects krill physiology (Ikeda, 1985; Quetin, Ross, & Clarke, 1994). Although the intensity of impacts from environmental change on krill is spatially heterogeneous, experimental and observational studies agree that a rise in SST beyond the threshold of krill survival (~4°C) will consistently result in high krill mortality (Constable et al., 2014; Hill et al., 2013; Murphy et al., 2007; Wiedenmann, Cresswell, & Mangel, 2008). Furthermore, due to the direct dependency of krill on primary productivity, both as adults and as juveniles, climate‐driven changes in chlorophyll are expected to affect krill directly through loss of food resources (Atkinson et al., 2006), given that their life cycles are synchronous with phytoplankton blooms (Clarke, 1988), while reductions in sea ice will affect krill indirectly through loss of habitat and associated ice algae (Arrigo, Dijken, & Pabi, 2008; Meiners et al., 2012).

In Models 1 and 2 we applied additional environmental forcing by including a climate–growth parameter, which was based on a statistical model (Atkinson et al., 2006) that relates experimentally validated increases in Antarctic krill length (mm.d^−1^) to SST (°C), and food availability indicated by chlorophyll‐a concentration (*CHL*, mg.m^−3^). Although there are a number of alternative models for evaluating Antarctic krill growth, few estimate growth as a function of temperature, which our literature review (Table S5) identified as a key determinant of krill survival (Wiedenmann et al., 2008). The model of Atkinson et al. (2006) has been used in several previous studies (Atkinson et al., 2008, 2009; Wiedenmann et al., 2008) to estimate krill Gross Growth Potential, which provides a measure of the ability of the habitat to support Antarctic krill growth, as follows:

(7) $$\begin{array}{cc} GR_{L,y}^{A} & =a^{\mathrm{GR}}+\left(b^{\mathrm{GR}}\cdot \ell^{\max}\right)+\left(c^{\mathrm{GR}}\cdot {\left(\ell^{\max}\right)}^{2}\right)+\frac{d^{\mathrm{GR}}\cdot \chi_{L,y}^{CHL,A}}{e^{\mathrm{GR}}+\chi_{L,y}^{CHL,A}}\\ & \;+\;f^{\mathrm{GR}}\cdot \chi_{L,y}^{TEMP,A}+g^{\mathrm{GR}}\cdot {\left(\chi_{L,y}^{TEMP,A}\right)}^{2} \end{array}$$

where *a^GR^, b^GR^, c^GR^, d^GR^, e^GR^, f^GR^*and *g^GR^*are constants and $\ell^{\max}$ is the maximum length of krill in mm, input from Atkinson et al. (2006) (Table S2). Note, because mean SSTs in Latitude band 40–50°S are above the estimated mortality threshold for krill, we constrained krill distribution (and associated consumption values for whales) to latitudes below 50°S (McLeod, Hosie, Kitchener, Takahashi, & Hunt, 2010). Model 3 excludes climate drivers, thus $GR_{L,y}^{A}=1$.

The krill population was assumed to be at deterministic equilibrium (corresponding to an absence of harvesting) at the start of the initial year of the NPZD model (1900). We calculated the spatial distribution of pre‐exploitation spawning biomass $B_{L,0}^{sp,A}$ of krill per Latitude and Area from maps of observed circumpolar distribution of Antarctic krill developed in Hill et al. (2013), which details individuals.m^−2^ within each 5° longitude by 2° latitude grid cell across the Southern Hemisphere (derived from Atkinson et al., 2008). We model interannual recruitment variability among regions using deviations from the Beverton‐Holt (Siegel, 1987, 2005), whereby residuals were computed based on the relative phytoplankton proportion in each Latitude and Area—that is, we assumed the variability in krill was driven by variability in phytoplankton ($\rho_{L,y}^{rel,A}$) (see Tulloch et al., 2017), as follows:

(8a) $$R\left(B_{L,y}^{sp,A}\right)=\left(\frac{4h\cdot R_{L,0}^{A}\cdot B_{L,y}^{sp,A}}{B_{L,0}^{sp,A}\left(1-h\right)+B_{L,y}^{sp,A}\left(5h-1\right)}\right)\cdot e^{\left(\varepsilon_{L,y}^{k,A}-{\left(\sigma_{R}\right)}^{2}/2\right)}$$

where *h* is the stock recruitment steepness parameter (from Kinzey et al. (2015)), $R_{L,0}^{A}$ is the amount of pristine recruitment in Area *A* Latitude *L*, $\varepsilon_{L,y}^{k,A}$ is the stock‐recruitment residual for krill in Latitude *L*of Area A and year *y*(which we set equal to $\rho_{L,y}^{rel,A}$) and $\sigma_{R}$ is the standard deviation of the log krill stock‐recruitment residuals (Table S2). We calculate $R_{L,0}^{A}$ based on the starting values for biomass trajectories (where $B_{L,0}^{sp,A}$ is input as described above), using the following equation:

(8b) $$R_{L,0}^{A}=B_{L,0}^{sp,A}/\sum_{a=1}^{z-1}w_{a}\exp\left(-M\cdot \left(z-2\right)\right)+w_{z}\frac{\exp\left(-M\cdot \left(z-1\right)\right)}{1-\exp\left(-M\right)}$$

The growth, abundance and spawning success of krill in Models 1 and 2 were thus influenced directly by both environmental variables (temperature, chlorophyll), whale predation, as well as the availability of prey for the krill themselves over time (Table 1). There is no feedback into the NPZD model from krill consumption to phytoplankton biomass, but our model incorporates feedback from whale consumption into krill biomass.

We used the relative phytoplankton value $\rho_{L,y}^{rel,A}$ as a proxy for copepod prey in our models, derived from the NPZD model outputs. This is consistent with previous research showing mesozooplankton productivity to be positively correlated with primary productivity (Friedland et al., 2012).

### 2.2 Predators (baleen whales)

We used delay‐difference equations to describe the whale dynamics, with seasonal time steps (see Tulloch et al. (2017) for detailed description of equations and parameter settings for whale dynamics without climate links). Summer dynamics were as follows:

(9a) $$\begin{array}{cc} N_{S1,y+1}^{j,A} & =\left(N_{S2,y}^{j,A+1}-H_{S2,y}^{j,A+1}\right)\cdot S^{j,A+1}\cdot f{\left(B_{y+1}^{A+1}\right)}^{j}\\ & \;+\;\left(N_{S2,y+1-T_{j}}^{j,A+1}\right)\cdot q^{j,A+1}\cdot \eta_{y+1-T_{j}}^{j,A+1}\cdot S_{\mathrm{juv}}^{*j,A+1}\cdot P^{j,A+1}\cdot f{\left(B_{y+1-T_{j}}^{A+1}\right)}^{j}\cdot {\left(S^{j,A}\cdot S^{j,A+1}\right)}^{T_{j}-1} \end{array}$$

and winter dynamics:

(9b) $$N_{S2,y+1}^{j,A}=\left(N_{S1,y+1}^{j,A-1}-H_{S1,y+1}^{j,A-1}\right)\cdot S^{j,A-1}$$

where $N_{S1,y}^{j,A}$ and $N_{S2,y}^{j,A}$ are numbers of female whale species *j,*area *A,*in year *y;* $H_{y,S1}^{j,A}$ and $H_{y,S2}^{j,A}$ are historical catches of female whales, species *j,*area *A,* in the summer (poles) S1 and winter (tropics) S2, respectively, assumed to occur as a pulse at the start of the season during time step *y*(input from IWC data, separated into each of the four areas according to the catch date and location, see Tulloch et al. (2017) for a detailed description of catch derivation)*;* $S^{j,A}$, $T^{j,A}$, $P^{j,A}$ and $q^{j,A}$ are the post‐first‐year 6‐month survival rate, average age at maturity (assumed to be 1 year more than the age at sexual maturity to account for average gestation period; computed from the Leslie matrix), maximum annual number of offspring and fraction of female calves (input from catches), respectively, of whale species *j* in area *A* (see Table S2); and $\eta_{y}^{j,A}$ is the density‐dependence term (based on Thomson, Butterworth, Boyd, and Croxall (2000) and Punt et al. (2016), see Tulloch et al. (2017) for detailed description of the density‐dependence calculation). Values of the population parameters such as survival rates for each species were fixed at the best available values (Table S2). For all models, we assumed prey availability affects the survival of baleen whales using a predator–prey interaction term $f{\left(B_{y}^{A}\right)}^{j}$ that links whale numbers to the relative abundance of phytoplankton and krill as follows:

(10) $$f{\left(B_{y}^{A}\right)}^{j}=\sum_{L}\nu_{L}^{j,A}\left(\tau_{L}^{j,A,krill}\cdot \frac{\alpha^{j}\cdot B_{L,y}^{sp,A}/B_{L,0}^{sp,A}}{\left(\alpha_{j}-1\right)+B_{L,y}^{sp,A}/B_{L,0}^{sp,A}}+\tau_{L}^{j,A,phy}\cdot \frac{\alpha^{j}\cdot \rho_{L,y}^{rel,A}}{\left(\alpha^{j}-1\right)+\rho_{L,y}^{rel,A}}\right)$$

where $\alpha^{j}$ is the prey interaction parameter for species *j* derived from $h^{\mathrm{pred}}$, the parameter that controls the shape (steepness) of the relationship between predator–prey net interaction outcome (Plagányi & Butterworth, 2012) and prey abundance (which is input, Table S2), as follows:

(11) $$\alpha =4h^{pred,j}/\left(5h^{pred,j}-1\right)$$

To build upon previous research (Tulloch et al., 2017), we included two‐way feedbacks in the models between whales and krill using the consumption term (described above), accounting for heterogeneity in the diet of the different whale species and defined $\tau_{L}^{j,krill}$ as the proportion of the diet of whale species *j* in Latitude *L* that is comprised of krill relative to phytoplankton diet proportion $\tau_{L}^{j,phy}$ (see Table S4). We computed the value of the predator–prey interaction term for each whale species in each Area and year by averaging over the interaction factors weighted by the relative spatial areas they feed in ($\nu_{L}^{j,A}$) and their preferred diet (krill vs. copepods). Future whale numbers for Models 1 and 2 were thus driven indirectly by changes in prey availability due to warming (Table 1), contrasting with Model 3 where climate drivers were removed from predator and prey dynamics.

**Figure 2:**
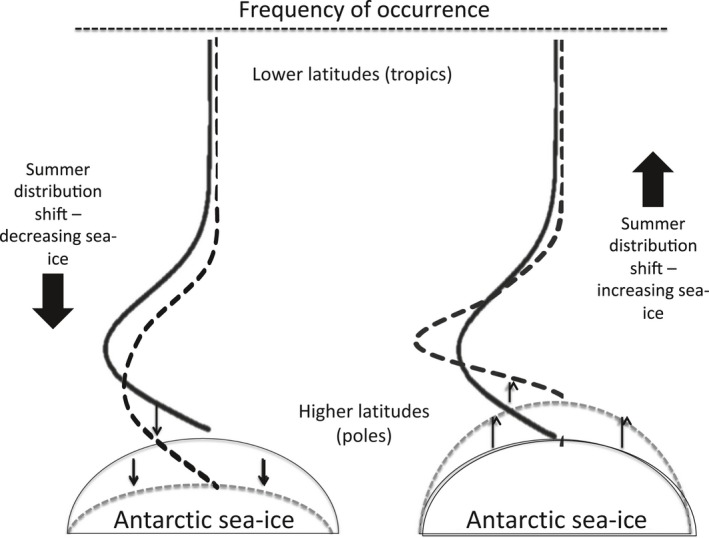
A schematic depiction of Southern Hemisphere whale migration highlighting postulated changes in migration extent. The bold black curves show the proportional distribution by latitude of one of the whale species, and dashed curves are hypothesized distribution shift due to changing sea‐ice extent in Antarctica, which is also identified by the dashed line

We also explore to what extent future climate change impacts may be lessened by whales shifting their distribution to better align with changing prey distributions. A separate set of equations were used in Model 2 to account for the relative favourability of environmental conditions encountered for whales based on the sea‐ice outputs of the coupled climate–NPZD model. Rather than explicitly modelling individual whale movement, we used a probability distribution function for whale species and each time step (although it was held constant over the historical period to 2012) to describe the relationship between whale distribution and sea‐ice extent and thickness. In the first instance, parameters of this relationship were estimated using $\nu_{L}^{j,A}$. A gamma distribution was derived for each historical whale species distribution from this information, and shape and rate parameters were estimated with respect to latitude. We assumed changes in sea ice affected relative favourability of environmental conditions encountered by whales, with modifications to the alpha shape parameter shifting each whale distribution north or south according to corresponding increases or decreases in sea ice (Figure 2). We used relative changes in sea‐ice thickness $i_{y}$as the multiplier so that each whale distribution at each Latitude *L* changes relative to those corresponding to starting or base ice conditions ($i_{2012}$). We held whale distributions prior to 2012 constant. Although the direction of change can be predicted with some certainty (see Figure 2), its extent remains uncertain and hence our approach provides an illustrative approximation only, but could be validated with fine‐scale data on the distribution of whales with respect to sea ice.

We fitted whale trajectories to an index of abundance from available surveys for the five whale species adjusted to represent female numbers only. Parameter uncertainty was explored in a previous model version (Tulloch et al., 2017), with best fits used to derive final input parameters for the models in this study. Sensitivity analyses were conducted to the input interaction parameter that controls the shape of the relationship between predator breeding success and prey availability (described in Plagányi & Butterworth, 2012, Tulloch et al., 2017) for each whale species. We also tested the sensitivity of whale projections to the distribution of whale feeding patterns and the availability of krill food source. We shifted the gamma shape parameter for whales 5° south, and then assumed some prey switching occurred by decreasing the proportion of krill consumed and increasing the proportion of alternative prey sources consumed (our copepod group in the model), such as has been recently observed for some whales in the mid‐latitudes (Findlay et al., 2017). Although all models provide plausible outputs, we focus our results primarily on Model 1, which includes defensible links between climate change and species based on the best available science under the current emissions scenario.

## 3 RESULTS

The primary climate model (Model 1) estimated less than 3% (*n* = 25,081, males and females) of the total preharvesting numbers of blue, fin, southern right and humpback whales remained across the Southern Hemisphere by the early 1970s, due to unsustainable catches of these species totalling over 1.3 million whales between 1890 and 2012 (Figure 1a,b). We exclude here minke whales, which were harvested only in relatively low numbers towards the end of the 20th century, and note southern right whales were already depleted by the end of the 19th century, with catches of this species in the 20th century almost exclusively taken by illegal Soviet whaling operations (Ivashchenko & Clapham, 2014), although small numbers of catches also occurred in the coastal waters of different countries. Estimates for the total abundance of these four species indicate that they are currently at 12% of their preharvesting levels, and numbers of all species are currently increasing. Although Model 1, which estimated 12 parameters for each whale species and included environmental forcing from temperature and phytoplankton production, but no sea‐ice effect, was the most parsimonious model based on the AIC (AIC = 47.9, Table S1), differences between model fits were not significant and historical model trajectories showed similar trends. This was expected given the small trend in temperature in the Southern Oceans during the historical period (Figure S3).

**Figure 3:**
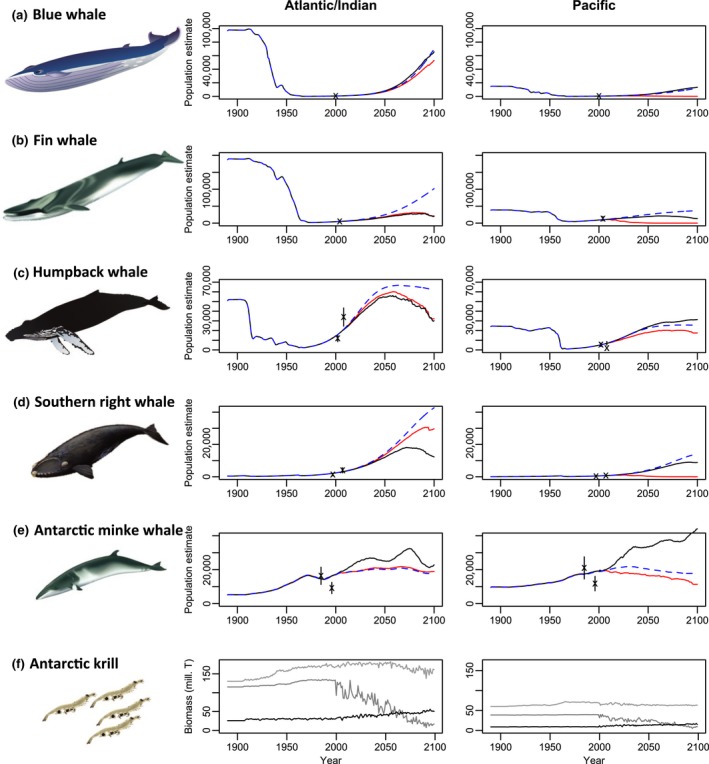
Model‐estimated whale population trajectories are shown for female population of (a) blue, (b) fin, (c) humpback, (d) southern right and (e) Antarctic minke whales in the Southern Hemisphere and (f) krill biomass predictions, for the Indian/Atlantic area (left) and the Pacific area (right). For whale population estimated (a–e), trajectories are shown for the preferred Model 1 linked to climate drivers (red line), Model 2 that includes sea‐ice links to whale distribution (black line) and the comparison with Model 3 that excludes climate drivers (blue dashed line). For whale trajectories (a–e), cross symbols show survey abundance observations and associated standard errors for the respective regions to which the model was fitted. Circumpolar estimates and fits are shown in the Supplementary (Figure S4). For krill biomass (f), we show predictions for latitudes 50–60°S (light grey), 60–70°S (grey) and 70–80°S, (black), for climate‐driven Models 1 and 2. There were no krill in latitudes 40–50°S. Note vertical axes have different scales [Colour figure can be viewed at http://wileyonlinelibrary.com]

Predictions from Model 1 show warming in the Southern Ocean under RCP8.5 will differentially affect southern baleen whale species, leading to population crashes of some populations and slowing the recovery of others by the end of this century (Figure 3). Despite continued predicted recovery of all species from depletion during the early 21st century, results demonstrate substantial reductions in total numbers of fin, blue and southern right whales by the end of this century. Vulnerable species such as fin whales that were depleted by >70% by historical harvesting are predicted to only be 5% of precommercial whaling numbers by 2100 across the Southern Hemisphere given projected changes in temperature, chlorophyll and primary productivity. Biggest declines are predicted in the Pacific, with populations of fin and southern right whales potentially becoming locally extinct by 2100, while blue whale numbers are predicted to be at <1% of precommercial whaling levels (Figure 3). Population declines were not as extreme in the Atlantic/Indian region, although model predictions show halted recovery for fin and southern right whales by the late 21st century. Although humpback whales were predicted to make a full recovery by 2050, numbers of these whales in the Pacific are predicted to halve by 2100. Minke whale numbers have increased rapidly during the last century, but growth is predicted to slow in the Atlantic/Indian region or even reverse in the Pacific over the next 100 years.

**Figure 4:**
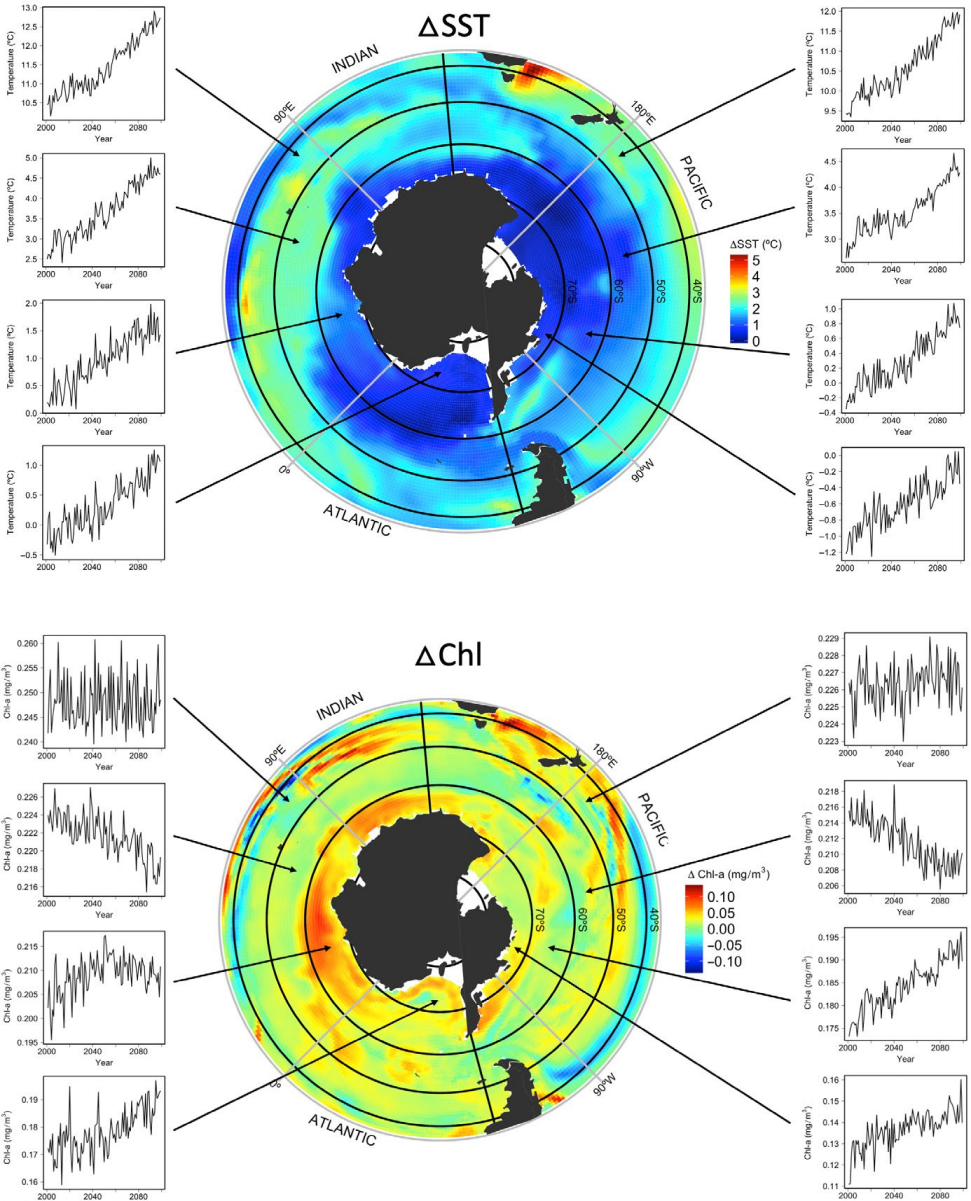
Changes in projected SST (top) and Chl‐a (bottom) from 2001 to 2100 from the coupled global climate–NPZD model. SST and Chl‐a change relative to starting value in 2001 is shown by the colour scale in each map, black circumpolar bands identify the four latitude bands used in the model (40–50°S, 50–60°S, 60–70°S and 70–80°S), thick black lines at 60°W and 130°E identify breaks between the two oceanic regions modelled. Change over time (*x* axis, years between 2001 and 2100) in SST and Chl‐a shown for each latitude band for Atlantic/Indian area (left graphs) and Pacific area (right graphs). Note the vertical axes have different scales [Colour figure can be viewed at http://wileyonlinelibrary.com]

Reduced krill biomass due to climate change combined with increased competition for krill prey by initially recovering whale populations are largely driving the modelled future declines in whale predators and krill prey (Figure 3). For southern right whales, however, declines were associated with changes in productivity and declines in chlorophyll (Figure 4) and subsequently their primary food of copepods in sub‐Antarctic mid‐latitude foraging grounds. Despite model trajectories showing historical increases in krill biomass to present day (Figure 3), declining overall trends are projected from now until the end of the century. The magnitude of the projected krill biomass decline was greater in the Pacific region (19% decline overall) than in the Atlantic (16% decline overall) by 2100. Spatial disaggregation of climate drivers and krill biomass into 10° latitude bands allowed evaluation of finer‐scale trends, with model results showing disparate spatial trends between latitudes exhibited by climate drivers and krill biomass over the next century (Figures 3f, 4). Although warming was predicted across all latitude bands, the greatest future warming by the end of the 21st century was predicted between 40 and 60°S. Changes in SST predicted in the climate–NPZD model were highest in the Atlantic/Indian region, with an average 2.5°C warming in 40–50° and 2.2°C warming in 50–60° (Figure 4), although in some areas of these oceans SSTs may increase by almost 5°C by 2100. The most rapid loss of krill biomass was predicted in the mid‐latitudes (50–60°S), where declines to <15% of the estimated starting biomass were predicted by 2100 (Figure 3f), due largely to projected warming in this latitude from ~2.5°C currently to >4°C by 2060 (Figure 4), considered the threshold for krill survival. Greater declines in krill of >85% of historical biomass in this latitude were predicted for the Atlantic/Indian region, compared to a 76% decline in the Pacific, however, greater increases were also predicted in the Atlantic/Indian highest latitudes around the Antarctic where krill biomass almost doubled by 2100. There were contrasting modest trends in Chl‐a in the different oceans and latitudinal bands, with some remaining unchanged and others showing increases or decreases (Figure 4). In particular, decreases in Chl‐a were observed between 50 and 70° by 2100 in the Atlantic/Indian, although in the Pacific, only the region between 50 and 60° showed decreases in Chl‐a, with continuing increases in Chl‐a across the latitudes 60 and 70° in the Pacific region over the next century.

Changing environmental conditions and associated spatial and temporal variability in krill biomass differentially affected whale numbers depending on their feeding distribution across latitudes and oceanic regions (Figure S2). Predicted declines in krill and copepod biomass in latitudes 50–60°S resulted in declining numbers of southern right, humpback and fin whales that feed predominantly in the mid‐latitudes. In contrast, increases in krill biomass around Antarctica in the highest latitudes (70–80°S, Figure 2f) resulted in concomitant increases in numbers of ice‐associated blue and minke whales in the Atlantic/Indian region (Figure 3a,e).

We ran alternative models with no climate forcing to test the sensitivity of whale population dynamics to intraspecific competition and changing prey availability, and of krill dynamics to predation versus changing environmental conditions. Although model fits to historical data were similar to the climate‐forced model (Table S1) implying that most historical changes reflect harvesting and whale recovery, predicted trajectories under more extreme environmental conditions diverged (Figure 3f). Recovery was predicted to continue for all whale populations throughout the 21st century when climate links were removed from the model. Importantly, the full recovery predicted for humpback whales by 2050 is strongly reversed in the climate‐forced model after 2050, particularly in the Atlantic/Indian region. Dramatic differences in predicted trajectories between models were observed for Pacific whale populations, which were predicted to make near complete recoveries when there was no climate forcing.

We also tested the sensitivity of whale projections to the distribution of whale feeding patterns and the availability of their krill food source. First, we projected whale abundance when the proportional distribution of mid‐latitude krill‐feeding whales (humpback, fin and southern right) was shifted 5° south to where krill density is currently greater, and we assumed some prey switching occurred by halving the proportion of krill consumed by whales in latitudes 50–60°S and increasing the corresponding proportion of alternative prey sources consumed (our copepod group in the model). Few major changes were observed in recovery trajectories for Pacific populations, but faster recovery for fin and blue whales was predicted in the Atlantic/Indian region, although this tapered off for fins by the end of the 21st century (Figure S5). Alternative krill carrying capacity values were also evaluated (Atkinson et al., 2009; Siegel, 2005), given variability in current circumpolar estimates, with the upper threshold of biomass estimates (>500 million tonnes (Atkinson et al., 2009, Siegel, 2005)) slowing the reduction in mid‐latitude krill biomass and slightly improving outcomes for whales in the Pacific. The sensitivity of the model to different forms of the functional relationship between predators and prey was examined by modifying the interaction parameter, but there was little change in future trajectory trends of each whale species. Our climate‐forced model projections demonstrate that the direct and indirect effects of changing environmental factors are much more influential in driving changes in krill and whale numbers than the specifics of the two‐way predation interaction between whales and krill alone.

**Figure 5:**
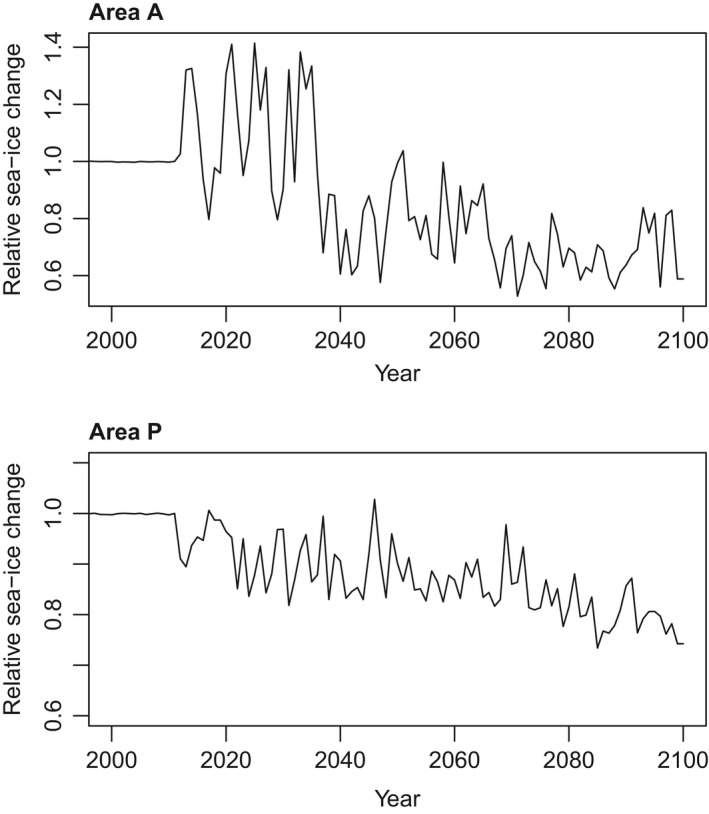
Mean projected 21st century sea‐ice extent change across latitudes 50–80°S in Area A (Atlantic/Indian) and Area P (Pacific), showing proportional change relative to sea‐ice extent in the year 2000

We ran an additional model investigating the adaptive capacity of whales to future sea‐ice change, based on whale energy expenditure and distribution. Concomitant with projected warming in Antarctic waters from the climate–NPZD model were projected changes in sea‐ice extent, with the greatest melting in our model predicted in the Atlantic/Indian sector (Figure 5). Whale model fits to the historical data were identical to the climate‐forced scenario as extent was fixed prior to 2012. Abundance of most whale populations benefitted from adapting their range to changing sea ice (Figure 3). Pacific whale populations benefitted the most from expansion of Antarctic ice‐free habitat when they were allowed to adapt to changing sea ice, with considerably slower declines predicted for southern right and fin whales than in the base model, and faster recovery in the case of humpback, blue and minke whales (Figure 3). Minke whales benefited the most from changing sea ice across both regions and southward shifts towards the poles, with rapid increases in numbers over the next century tracking increasing krill biomass trends in the highest latitudes (70–80°S). These modelled increases in krill biomass in the highest polar latitudes were likely a result of increases in Chl‐a, which may be moderating the concomitant warming of up to 1.8°C in this region (Figure 4). In contrast, southern right whales in the Atlantic/Indian did not benefit shifting distribution given changing sea ice due to their predominant distribution in mid‐latitudes where prey declines are predicted.

## 4 DISCUSSION

The recovery of baleen whales in the past few decades following the cessation of >200 years of intense whaling in the Southern Ocean exemplifies the benefits of modern conservation protection measures, such as the restrictions on commercial whaling implemented by the IWC, to reduce human pressures on species of conservation concern. Earlier research predicted that baleen whale populations previously depleted by historical whaling would continue to recover (Mori & Butterworth, 2006; Tulloch et al., 2017), but these studies did not consider the impact of anthropogenic climate change on food availability in the Southern Ocean. We found the long‐term and potentially irreversible changes to physical processes and the marine environment that are expected with future climate change threaten the recovery of these whale species. Our coupled climate–biological models predicted future negative impacts of climate change for krill and all whale species, although the magnitude of future climate change impacts on whales differs among populations. Despite demonstrated recovery of whales throughout the late 20th century after depletion from historical whaling, we show strongly negative future trajectories for Pacific populations of blue, fin and southern right whales, with potential for extinction. There were also large declines in fin and humpback whales in the Atlantic/Indian oceans. These trajectories highlight conservation concerns for local populations of fin and blue whales, with both species currently listed on the IUCN Red List as endangered (Reilly, Bannister, & Best, 2008a). Our findings suggest that whales feeding in mid‐latitude areas may be more heavily impacted by climate‐driven changes in prey availability than those that are distributed further south. Although the impact of climate change on ecological processes is difficult to quantify, we used the latest information about climate impacts on lower trophic levels (see Table S5) to parameterize our models in the Southern Ocean and validate key links between climate change drivers and low trophic krill prey (Table S5). Our models substantially extend previous research (Tulloch et al., 2017) by including both two‐way predator–prey interactions and also linking multiple climate drivers (i.e. SST, chlorophyll and sea ice) to whales and their krill and copepod prey. As such, they provide the first holistic predictions of the combined effects of multiple future climate stressors on interacting krill and whale species throughout the Southern Hemisphere.

Climate change may shape the survival of marine species both through temporal or spatial mismatches in trophic interactions and the seasonal timing of prey availability (Anderson et al., 2013; Cushing, 1974), as well as from changing environmental conditions, such as the impact of warming on species distribution and migration patterns (Lloyd, Plagányi, Weeks, Magno‐Canto, & Plagányi, 2012; Sharp, 2003). Our findings highlight the possible effects of spatial mismatches in krill and copepod prey availability. Whale species predominantly feeding in mid‐latitudes (40–60°S), such as humpback, fin and southern right whales, were more heavily impacted than those distributed further south because of predicted declines in krill and copepod prey in mid‐latitudes. This highlight that areas around the Antarctic circumpolar current are highly vulnerable to climate‐driven changes (Hill, Murphy, Reid, Trathan, & Constable, 2006), and supports recent evidence of the direct cause–effect of the climate–krill–whale relationship for southern right whales (Seyboth et al., 2016).

Our model results suggest that increased competition for krill prey by initially recovering whale populations over the next 100 years combined with predicted increases in minke whale numbers may be driving some of the projected further declines in both krill prey and whale predators. Minke whales responded earlier to increasing levels of krill abundance because the whale–krill functional relationship is most sensitive for minkes based on historical dynamics (see Table S2) and their populations grow faster than the bigger whale species (Taylor, Chivers, Larese, & Perrin, 2007). Thus, minke whales track krill biomass declines in the latter part of the 21st century, particularly in the Pacific mid‐latitudes where minke populations are abundant. Environmental change is likely to have a large effect on Antarctic predator–prey interactions and thus on energy transfer in marine systems (also see Trivelpiece et al., 2011).

We found fairly minor differences in historical whale trajectories and hence in the quality of model fits between our models. This was because historically the changes in temperature in the southern oceans have been small and hence there are no historical analogues to validate the influence of rising temperatures on krill and whales at large scales. Climate change is expected to accelerate in the future, with an increase in atmospheric CO2 of another 560 ppm expected by the end of the century (940 ppm) under the RCP8.5 scenario, and hence the model predictions for whale populations start to diverge. Additionally, there are too few survey and other data available to reliably distinguish between alternative models attempting to explain recent changes in whale populations due to environmental changes over the past two–three decades (the period for which data are available from our model and which includes a slow rise in SST) (Punt, 2014; Punt, Bando, Hakamada, & Kishiro, 2014). Our historical estimates of total circumpolar whale numbers are consistent with previous estimates (Mori & Butterworth, 2006; Tulloch et al., 2017). Hence, although we did not find a statistically significant difference between our no‐climate and climate‐forced historical model, this also means that including environmental drivers and assuming that these influenced whale dynamics historically is not inconsistent with past observations and provides a comparably good alternative explanation of observed changes over recent years. Other evidence from studies of whale ecology suggests the model of climate effects on whales via their prey (Model 1) is most realistic. Whale populations historically may have responded fairly dramatically to changes in krill due to the massive declines in whales from historical harvesting (Ainley et al., 2007; Surma, Pakhomov, & Pitcher, 2014), although this is still subject to debate (Ballance et al., 2006). Evidence for strong density dependence in response to prey availability in these whale populations is supported further by demographic data documenting changes in the age at first reproduction (e.g. minke whales (Masaki, 1979) and fin whales (Lockyer, 1972)). Given these uncertainties, future research into the strength of feedbacks between whale population size and krill numbers is therefore important for predicting the response of whales to climate change.

Model trajectories show substantially slower rates of increase in whale populations over the next 50 years, in some cases reduced by one third to those of the climate‐decoupled trajectories (Model 3), leading in some cases to population crashes not observed in models that ignore multiple climate impacts (e.g. Mori & Butterworth, 2006, Tulloch et al., 2017). Differences between our projections and earlier projections for baleen whales in the Scotia sea (Klein et al., 2018) highlight the importance of some key processes that will determine future whale population trends. Projections from the Scotia Sea were more optimistic than the trends projected here, which covered the entire Southern Ocean, suggesting that some subpopulations may benefit from climate change, but overall there will be declines in whale abundance. The difference between projections for whale population trends that aggregate baleen whales into a single functional group (Klein et al., 2018) versus modelling whale species as potential competitors for krill (this study) highlights the importance of interspecific differences in how whale species respond to environmental conditions, and competitive interactions among whale species in determining future recovery trajectories.

This study fills an important knowledge gap concerning how baleen whales might respond to a changing climate, including examining how phenotypic plasticity may improve recovery for certain species. We highlight potential benefits for some whale populations, particularly those ice‐associated species distributed across high latitudes such as blue and minke whales, if they have the capacity to adapt and change their feeding patterns given changing sea ice in the Antarctic and a shifting prey base. This is because of projected increases in productivity and low trophic prey biomass around Antarctica, compared to large declines in their primary krill prey around the Antarctic circumpolar current further north. Such phenotypic plasticity has already been observed in the Northern Hemisphere, with changes in phenology in response to ocean warming demonstrated by fin and baleen whales in the Gulf of St Lawrence Canada (Ramp, Delarue, Palsbøll, Sears, & Hammond, 2015), and northward shifts in distribution of blue and fin whales around Iceland as a response to changes in the marine environment (Víkingsson et al., 2015).

The climate predictions used in this study are broadly consistent with other climate models and observations. The greatest future warming by the end of the 21st century was predicted in the Atlantic/Indian region, supporting recent findings of rapid climate change and sea‐ice melt already observed in the West Antarctic Peninsula (Mulvaney et al., 2012; Vaughan et al., 2003). Similarly, model predictions of sea‐ice melt are consistent with previous Antarctic temperature‐index melt modelling (Lee et al., 2017) and observational records of the greatest melting to date off the West Antarctic Peninsula (Meredith & King, 2005). Krill declines predicted by the model align with recent experimental and observational research showing slower krill growth and higher mortality at warmer temperatures (Kawaguchi et al., 2013, 2011) and are consistent with recent models that predict future declines in krill in the Scotia Sea under an RCP8.5 warming scenario (Klein et al., 2018).

Our study has several inherent assumptions and uncertainties inherent in our approach. First, the global climate–NPZD model used in this study uses the highest greenhouse emission scenario (RCP8.5 (Peters et al., 2013)) to force changes in productivity. As global emissions are currently tracking this emissions trajectory, this scenario indicates the plausible extent of impacts that could be seen on whales and krill if current CO_2_ emissions are not reduced (Sanford et al., 2014). Our goal here was to investigate whether whale recovery might be compromised by climate change, rather than present a range of possible future scenarios given changing emissions. However, dramatic differences in our findings of continuing recovery for all whale populations throughout the 21st century when climate links were removed from the model, compared to population crashes once climate impacts were included on krill and whales, provide insight into the benefits to whale populations if global emissions were reduced. Importantly, our results provide an early warning of the plausible future population changes to be expected for whales under the current emission scenario. This evidence is important to proactively inform strategic thinking regarding future sustainable krill catch levels. Future monitoring could be used to validate or negate our predictions, although this would require ongoing surveys of whale populations that employ novel nonlethal techniques for monitoring changes in key demographic parameters such as age at first reproduction.

Second, outputs from the sea‐ice–linked model are only a first approximation to the types of direct impacts that habitat change and warming might have on whales. Other possible adaptive mechanisms warrant future investigation, including whether predicted warming in lower latitudes where migratory whales breed might increase the chance of juvenile survival due to range contractions, and decreased energy expenditure during migrations between feeding and breeding grounds (Cooke, Rowntree, & Payne, 2003; Leaper et al., 2006; Walther et al., 2002). We also did not explicitly model krill–ice dynamics because of insufficient data to quantify the relationships, but did consider their observed temperature preferences. Expected decreases in sea ice in some regions of the Antarctic may reduce krill survival more than we have incorporated here due to their reliance on ice algae as a food source in high latitudes (Flores et al., 2012). However, other macrozooplankton species could move south from more temperate waters and replace Antarctic krill, and this could be explored in future research using alternative scenarios of krill distribution shift. Furthermore, research shows salps dominate Antarctic marine ecosystems during poor krill years (Atkinson et al., 2004), and the effect of the dominance of salps with sea‐ice reduction due to warming could be explored in future models. This model helps reduce some unknowns in krill–climate dynamics, despite a paucity of data, but future work would benefit from using more accurate survey information and improved experimental understanding of the responses of krill to multiple climate impacts. On the other hand, our projections may be too conservative if as Steffen et al. (2018) note, self‐reinforcing feedbacks push the planet on a much more severe “hothouse trajectory”, with melting of the East Antarctic ice sheet identified as one potential tipping element.

Third, given the complexities of modelling whale consumption over a large geographic area and based on limited information, we used the intermediate complexity approach to represent the overall net outcome of the predator–prey interactions. For simplicity we assumed predation by other species on krill remained constant, but these species may exert a strong influence on prey biomass in Southern Ocean ecosystems. The abundance of species such as seals that prey on krill has been posited to have increased in the wake of whale depletion during the mid 20th century (Mori & Butterworth, 2006). We did not include other krill predators (e.g. seabirds, seals, penguins, fish, jellyfish and squid) due to the lack of information on their abundance. However, competition from seals is likely to make krill declines worse, therefore our results for whales that feed in the same region as seals may overestimate their capacity to recover from historic declines (Tulloch et al., 2017). This work provides the basis for extending the model from focusing on krill, copepods and whales to other important members of the ecosystem to explore further future competition scenarios. Last, commercial catches of krill were not included in the model because current catches are low in proportion to the overall estimated biomass of krill across the Southern Ocean (Nicol, Foster, & Kawaguchi, 2012). However, krill catches are expected to increase in the future (Nicol et al., 2012), and could further hamper recovery of whales. Exploring how krill fisheries impact whale recovery is a priority for further research (e.g. Klein et al., 2018).

Finally, we used deterministic model projections and a range of sensitivity tests to provide a first approximation of the likely impact of climate on future population trends. We acknowledge that natural variability is important too in these systems, and that stochastic simulations would enable a more in‐depth analysis of extinction risk. However, considering the already large uncertainty associated with predicting the influence of climate change on whale dynamics we focused on a first‐order approximation. This is also because there is additional spatial variability in krill population dynamics that operates at a finer scale than the broad scale we used in our model. Furthermore, given the different historical recovery rates shown by subpopulations of some species such as southern right and humpback whales (Jackson et al., 2016), heterogeneous future recovery and responses to environmental drivers and changes in prey at the subpopulation level is likely, and this could be tested in future work using a finer spatial resolution.

There are three potential options to lessen future risks to both vulnerable whale species and krill. First, greenhouse gas emissions could be reduced. With global emissions tracking the highest greenhouse gas emissions scenario (RCP8.5 (Peters et al., 2013)), emission reductions will slow the trophic impacts of warming on Antarctic ecosystems, such as changes in krill biomass. It is beyond the year 2030–2040 where the projected trajectories of some whale populations start to diverge in the vulnerable Pacific region given climate change. There is still time to reduce emissions and minimize the impact of climate change on recovering whale populations. This will also have direct implications for maintaining commercial krill fisheries in the Southern Ocean, particularly given the expected future expansion of krill fisheries, which is likely to further compromise the recovery of baleen whales. Second, consideration of the climate‐driven trophic impacts on krill could inform setting sustainable catch limits in the future (Kawaguchi, Nicol, & Press,^2009^), which would improve resilience of dependent predators such as whales (Trivelpiece et al., 2011). This is particularly important considering the expected future expansion of krill fisheries (Nicol et al., 2012), and projected warming to a level that exceeds the thermal tolerance of krill, leading to reduced catch potential in the Atlantic/Indian region where krill fisheries and many whales are currently concentrated (Constable, 2002; Hewitt, Watters et al., 2004; Kock, Purves, & Duhamel, 2006). The temperature threshold in our krill growth model resulted in a stepwise (nonlinear) more negative impact on krill, highlighting the need to avoid extreme temperature thresholds to maintain a safe operating space within which Southern Ocean ecosystems can continue to recover and thrive (Rockström et al., 2009b; Scheffer et al., 2015). This is consistent with the growing consensus towards a “2°C guardrail” approach, whereby the rise in global mean temperature is contained to no more than 2°C above the preindustrial level (Rockström et al., 2009a). Last, there is the potential to reduce nonclimate stressors such as fishing gear interactions, ship strikes, noise pollution and commercial whale harvest, all of which reduce whale numbers or negatively affect whale fitness (Clapham, 2016; National Academies of Sciences,^2017^). Our findings show Pacific blue, fin and southern right whales are the most at risk, and continued monitoring of these populations is needed. Given that both humpback and southern right whales, currently listed as Least Concern by the IUCN Red List (Reilly et al., 2008a, 2008b, 2013), were predicted to decline in numbers, re‐evaluation of these taxa against the Red List criteria might be warranted in the near future, to higher risk categories (Akçakaya, Butchart, Mace, Stuart, & Hilton‐Taylor, 2006; Van Der Hoop et al., 2013). However, without immediate reduction in emissions to reduce global warming, the success of other regional management actions may be limited (Simmonds & Eliott, 2009; Simmonds & Isaac, 2007).
