# Supplementary material for: Future recovery of baleen whales is imperiled by climate change
Source: Glob Chang Biol. 2019 Feb 26;25(4):1263–81. doi: 10.1111/gcb.14573 (PMC6850638; doi:10.1111/gcb.14573)
Supplement: Supplementary file 1 [file GCB-25-1263-s001.docx]

**Supplementary Materials for**

**Future recovery of baleen whales is imperiled by climate change**

This file includes:

**Figs. S1 to S5**

**Tables S1 to S5**

**Supplementary references**

**
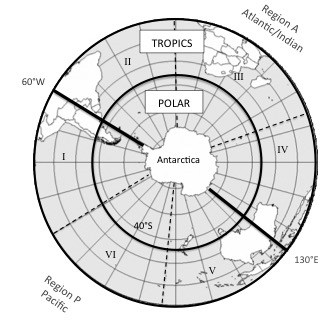
**

**Figure S1.** The four spatial areas used in the model, with labels and bold lines defining the separation between polar (40°–80°S) and tropics (0°–40°S) and Pacific (130°E–60°W) and Atlantic/ Indian (60°W–130°E) regions. For reference purposes, the plot also shows the six IWC whale stock management areas (dashed lines, roman numerals)

**Figure S2**. Base case whale distribution across latitude bands for summer feeding months, with the left graph highlighting spread of catches across the Atlantic/Indian region for each species, and right graph highlighting spread of catches in the Pacific region (see Tulloch *et al.* (2017)). A gamma distribution has been fitted to the observed catch proportions.


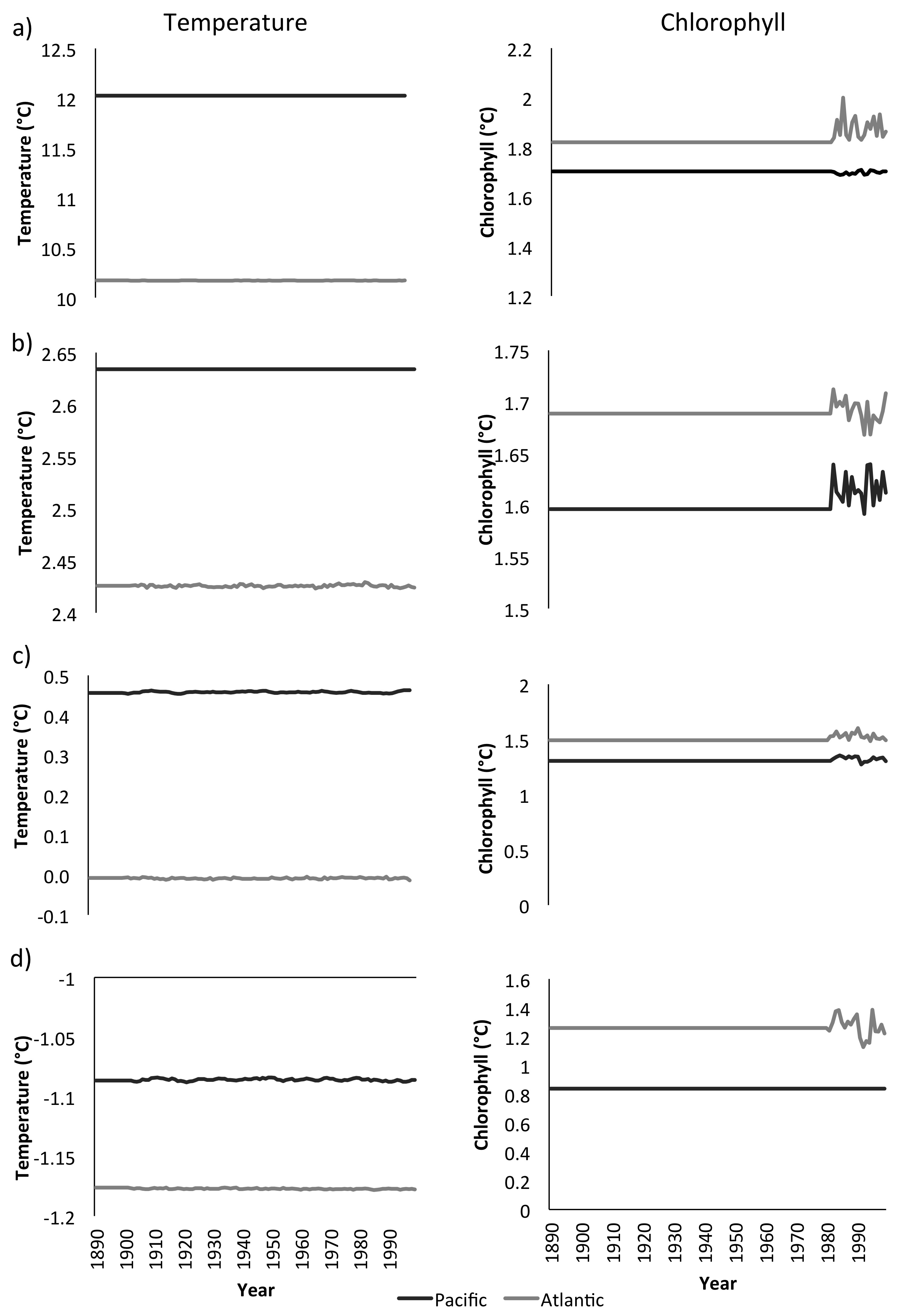


**Figure S3**. Sea-surface temperature and chlorophyll inputs into the MICE model from the global climate-linked NPZD model for the years 1890 to 1999.

**Figure S4.** Model-estimated circumpolar whale population trajectories are shown for female population of blue, fin, humpback, southern right, and Antarctic minke whales in the Southern Hemisphere, showing full trajectory (left) and “zoomed in” trajectories identifying model fits (right). Trajectories are shown for Model 1 linked to climate drivers (red line), Model 2 that includes sea-ice links to whale distribution (black line) and the comparison with Model 3 that excludes climate drivers (blue dashed line). Cross symbols show survey abundance observations and associated standard errors for the respective regions to which the model was fitted. Note vertical and horizontal axes have different scales.

**Figure S5.** Trajectories of female whale populations of sensitivity tests compared to Model 1 (red line): increasing krill carrying capacity (black line), exploring sensitivity to alternative assumptions regarding predator-prey interaction strength and form (grey line), and shifting whale distribution southwards to more favourable environmental conditions and changing feeding preference modifications (black dashed line).

**Table S1.** Summary of best-fit model with two-way interactions (Models 1 and 2), compared to Model 3 with no climate forcing. The Hessian-based 90% confidence intervals are shown for estimated parameters. The lower part of the table shows the negative log likelihood contributions.

| Model estimates | *Model 1*  *Base Case climate-forcing* | | | *Model 2*  *Climate-forcing adding sea-ice* | | | *Model 3*  *Excluding climate-forcing* | | |
| --- | --- | --- | --- | --- | --- | --- | --- | --- | --- |
| Species | *Estimated value* | *Lower 90% conf. int.* | *Upper 90% conf. int.* | *Estimated value* | *Lower 90% conf. int.* | *Upper 90% conf. int.* | *Estimated value* | *Lower 90% conf. int.* | *Upper 90% conf. int.* |
| *K ^b,A^* | 96939 | 96854 | 97056 | 96960 | 96856 | 97053 | 96934 | 96854 | 97056 |
| *K ^b,P^* | 14789 | 14763 | 14814 | 14781 | 14756 | 14806 | 14786 | 14762 | 14812 |
| *K ^f,A^* | 187290 | 186805 | 187621 | 187360 | 186993 | 187808 | 187350 | 186992 | 187809 |
| *K ^f,P^* | 38526 | 38227 | 38821 | 38508 | 38226 | 38821 | 38514 | 38227 | 38821 |
| *K ^h,A^* | 50926 | 50837 | 51002 | 50934 | 50837 | 51002 | 50932 | 50837 | 51002 |
| *K ^h,P^* | 24196 | 24099 | 24296 | 24171 | 24076 | 24271 | 24174 | 24076 | 24271 |
| *K ^r,A^* | 50870 | 50438 | 51303 | 50896 | 50488 | 51355 | 50863 | 50439 | 51302 |
| *K ^r,P^* | 16661 | 16499 | 16823 | 16666 | 16504 | 16829 | 16660 | 16499 | 16823 |
| $\vartheta^{A}$ | 0.009 | 0.001 | 0.006 | 0.009 | 0.003 | 0.014 | 0.009 | 0.005 | 0.014 |
| $\vartheta^{P}$ | 0.004 | 0.001 | 0.007 | 0.004 | 0.0001 | 0.008 | 0.004 | 0.002 | 0.009 |
| *K ^m,A^* | 52030 | 50675 | 53466 | 50513 | 49192 | 51871 | 52013 | 50625 | 53413 |
| *K ^m,P^* | 96980 | 94122 | 99873 | 93370 | 90632 | 96128 | 95612 | 92827 | 98470 |
| *Likelihood* | *Fit* | *Sigma* | *Q* | *Fit* | *Sigma* | *Q* | *Fit* | *Sigma* | *Q* |
| ‘-lnL:BlueTot | -3.946 | - | - | -3.959 | - | - | -3.942 | - | - |
| ‘-lnL:Blue_1981_ | -0.724 | 0.403 | 1.000 | -0.732 | 0.403 | 1.000 | -0.722 | 0.403 | 1.000 |
| ‘-lnL:Blue_1988_ | -0.435 | 0.489 | 1.000 | -0.435 | 0.489 | 1.000 | -0.434 | 0.489 | 1.000 |
| ‘-lnL:Blue_1996_ | -0.701 | 0.403 | 1.000 | -0.704 | 0.403 | 1.000 | -0.701 | 0.403 | 1.000 |
| ‘-lnL:Blue_1998_ | -0.200 | 0.724 | 1.000 | -0.201 | 0.724 | 1.000 | -0.199 | 0.724 | 1.000 |
| ‘-lnL:BlueI_2000_ | -0.945 | 0.385 | 1.000 | -0.945 | 0.385 | 1.000 | -0.944 | 0.385 | 1.000 |
| ‘-lnL:BlueP_2000_ | -0.942 | 0.385 | 1.000 | -0.942 | 0.385 | 1.000 | -0.941 | 0.385 | 1.000 |
| ‘-lnL:FinTot | -2.775 | - | - | -2.750 | - | - | -2.771 | - | - |
| ‘-lnL:Fin_1974_ | -0.666 | 0.385 | 1.000 | -0.658 | 0.385 | 1.000 | -0.664 | 0.385 | 1.000 |
| ‘-lnL:Fin_1983_ | -0.954 | 0.385 | 1.000 | -0.953 | 0.385 | 1.000 | -0.954 | 0.385 | 1.000 |
| ‘-lnL:FinI_2004_ | -0.712 | 0.472 | 1.000 | -0.710 | 0.472 | 1.000 | -0.712 | 0.472 | 1.000 |
| ‘-lnL:FinP_2004_ | -0.443 | 0.472 | 1.000 | -0.429 | 0.472 | 1.000 | -0.441 | 0.472 | 1.000 |
| ‘-lnL:HumpTot | -3.186 | - | - | -3.095 | - | - | -3.232 | - | - |
| ‘-lnL:Hump_1987_ | -0.872 | 0.349 | 1.000 | -0.887 | 0.349 | 1.000 | -0.863 | 0.349 | 1.000 |
| ‘-lnL:Hump_1999_ | 0.735 | 0.349 | 1.000 | 0.763 | 0.349 | 1.000 | 0.713 | 0.349 | 1.000 |
| ‘-lnL:HumpI_2002_ | -0.836 | 0.284 | 1.000 | -0.830 | 0.284 | 1.000 | -0.840 | 0.284 | 1.000 |
| ‘-lnL:HumpP_2002_ | -1.243 | 0.284 | 1.000 | -1.241 | 0.284 | 1.000 | -1.234 | 0.284 | 1.000 |
| ‘-lnL:HumpI_2008_ | 0.349 | 0.275 | 1.000 | 0.420 | 0.275 | 1.000 | 0.317 | 0.275 | 1.000 |
| ‘-lnL:HumpP_2008_ | -1.319 | 0.284 | 1.000 | -1.320 | 0.284 | 1.000 | -1.324 | 0.284 | 1.000 |
| ‘-lnL:RightTot | -1.199 | - | - | -0.994 | - | - | -1.207 | - | - |
| ‘-lnL:Right_1920_ | -0.497 | 0.275 | 1.000 | -0.492 | 0.275 | 1.000 | -0.500 | 0.275 | 1.000 |
| ‘-lnL:Right_2009_ | 1.182 | 0.275 | 1.000 | 1.188 | 0.275 | 1.000 | 1.181 | 0.275 | 1.000 |
| ‘-lnL:RightI_2007_ | -0.745 | 0.530 | 1.000 | -0.743 | 0.530 | 1.000 | -0.744 | 0.530 | 1.000 |
| ‘-lnL:RightP_2007_ | -0.947 | 0.530 | 1.000 | -0.947 | 0.530 | 1.000 | -0.947 | 0.530 | 1.000 |
| ‘-lnL:MinkeTot | -2.920 | - | - | -2.529 | - | - | -2.795 | - | - |
| ‘-lnL:Minke_1988_ | -0.790 | 0.530 | 1.000 | -0.735 | 0.530 | 1.000 | -0.763 | 0.530 | 1.000 |
| ‘-lnL:Minke_2012_ | -0.866 | 0.385 | 1.000 | -0.570 | 0.385 | 1.000 | -0.753 | 0.385 | 1.000 |
| ‘-lnL:MinkeI_1985_ | -0.780 | 0.385 | 1.000 | -0.726 | 0.385 | 1.000 | -0.770 | 0.385 | 1.000 |
| ‘-lnL:MinkeP_1985_ | -0.634 | 0.340 | 1.000 | -0.547 | 0.340 | 1.000 | -0.594 | 0.340 | 1.000 |
| ‘-lnL:MinkeIP_1996_ | 0.149 | 0.337 | 1.000 | 0.048 | 0.337 | 1.000 | 0.084 | 0.337 | 1.000 |
| Total likelihood (whales) | -14.026 |  |  | -13.327 |  |  | -13.946 |  |  |
| No. est. pars | 12 |  |  | 12 |  |  | 12 |  |  |
| AIC | 47.948 |  |  | 49.346 |  |  | 48.108 |  |  |

**Table S2**. Fixed input values for variables used in the base case whale and krill models. The input parameters shown in italics are base case values, with *S***^j,A^* calculated using Leslie Matrix equations (see Tulloch et al. 2017), *h^pred^* fixed based on sensitivity tests for the best-fit model, and *q^j,A^* derived from a combination of catch data and the literature (see Tulloch et al. 2017).

| **Whale species** | $q^{j,A}$ | ** | $h^{pred,j}$ | $T^{j,A}$ | $\lambda^{j,A}$ | $S^{j,A}$ | $P^{j,A}$ |
| --- | --- | --- | --- | --- | --- | --- | --- |
|  | Fraction of female calves | Juvenile survival rate (per year) | Predator-prey interaction steepness (0-1) | Ave. age at maturity (years) | Max. intrinsic growth rate | 6-monthly survival rate (per year) | Max. annual number of offspring |
| **Blue A** | 0.473 | 0.109 | 0.700 | *6* | *1.082* | *0.980* | *0.430* |
| **Blue P** | 0.474 | 0.109 | 0.700 | *6* | *1.082* | *0.980* | *0.430* |
| **Fin A** | 0.490 | 0.130 | 0.700 | *6* | *1.040* | *0.975* | *0.446* |
| **Fin P** | 0.480 | 0.132 | 0.700 | *6* | *1.040* | *0.975* | *0.446* |
| **Hump A** | 0.540 | 0.206 | 0.700 | *5* | *1.110* | *0.960* | *0.424* |
| **Hump P** | 0.530 | 0.210 | 0.700 | *5* | *1.110* | *0.960* | *0.424* |
| **Right A** | 0.470 | 0.070 | 0.900 | *6* | *1.073* | *0.990* | *0.321* |
| **Right P** | 0.470 | 0.081 | 0.900 | *6* | *1.073* | *0.990* | *0.275* |
| **Minke A** | 0.560 | 0.124 | 0.500 | *10* | *1.080* | *0.960* | *0.833* |
| **Minke P** | 0.560 | 0.124 | 0.500 | *10* | *1.080* | *0.960* | *0.833* |
| **KRILL** | | | | | | | |
| **Age-structured model values** | ***M*** |  | *$t_{0}$* | ***z*** | ***h*** | $\sigma^{R}$ |  |
|  | Time-invariant natural mortality rate | Max. krill length (mm) | Growth rate (annual) | Largest age (years) | Stock-recruitment steepness | Stnd. dev. of log krill stock-recruitment residuals |  |
|  | 1. 26 | 40.0 | 0.072 | 10 | 0.99 | 0.9 |  |
| **Growth parameter values** | $a^{GR}$ | $b^{GR}$ | $d^{GR}$ | $e^{GR}$ | $f^{GR}$ |  |  |
|  | -0.066 | 0.00674 | 0.377 | 0.321 | 0.013 | 40.0 |  |

**Table S3.** Consumption input parameters

| **Whale species** | *C (tonnes)* | *References* |
| --- | --- | --- |
| **Blue** | 350.04 | Ratnarajah *et al.* (2016) |
| **Fin** | 199.68 | Ratnarajah *et al.* (2016) |
| **Humpback** | 94.20 | Ratnarajah *et al.* (2016) |
| **Southern Right** | 18.00 | Perrin and Wursig (2009) |
| **Minke** | 36 | Tamura *et al.* (1997), Tamura and Konishi (2006), Tamura and Konishi (2009) |

**Table S4.** Consumption proportion of a) krill and b) phytoplankton/copepods across latitudes by whale population, derived from Tulloch *et al.* (2017).

| a) |  | Latitude | | | |
| --- | --- | --- | --- | --- | --- |
| Area | Whale populations | 40-50°S | 50-60°S | 60-70°S | 70-80°S |
| Atlantic | Blue | 0 | 1 | 1 | 1 |
|  | Fin | 0 | 0.8 | 1 | 1 |
|  | Humpback | 0 | 0.82 | 1 | 1 |
|  | Right | 0 | 0.5 | 0.5 | 0 |
|  | Minke | 0 | 0.94 | 1 | 1 |
| Pacific | Blue | 0 | 1 | 1 | 1 |
|  | Fin | 0 | 0.8 | 1 | 1 |
|  | Humpback | 0 | 0.82 | 1 | 1 |
|  | Right | 0 | 0.5 | 0.5 | 0 |
|  | Minke | 0 | 0.94 | 1 | 1 |

| b) |  | Latitude | | | |
| --- | --- | --- | --- | --- | --- |
| Area | Whale populations | 40-50°S | 50-60°S | 60-70°S | 70-80°S |
| Atlantic | Blue | 1 | 0 | 0 | 0 |
|  | Fin | 1 | 0.2 | 0 | 0 |
|  | Humpback | 1 | 0.18 | 0 | 0 |
|  | Right | 1 | 0.5 | 0.5 | 0 |
|  | Minke | 1 | 0.06 | 0 | 0 |
| Pacific | Blue | 1 | 0 | 0 | 0 |
|  | Fin | 1 | 0.2 | 0 | 0 |
|  | Humpback | 1 | 0.18 | 0 | 0 |
|  | Right | 1 | 0.5 | 0.5 | 0 |
|  | Minke | 1 | 0.06 | 0 | 0 |

**Table S5**. Summary of known direct responses of biota to changes in physical parameters in Antarctica and the Southern Ocean. The symbols used in the table denote a positive (+) or negative (-) direct effect of a change in the physical variable on *E. superba* (see text for details as to the reasons). ‘?’ indicates where there may be a response but the direction is uncertain, i.e. the result may be variable in space, time or for specific taxa or the evidence is unequivocal, or the response is not yet fully understood.

|  | Temperature increase | Sea-ice extent, thickness and duration | Increased CO_2_, ocean acidification | Increased UV and irradiance |
| --- | --- | --- | --- | --- |
| Mechanism for environmental change | Increasing atmospheric and oceanic temperatures due to global warming. Regional differences - mid‐winter surface atmospheric temperatures have increased in WAP by 6°C (>5 times the global average) in past 50 years (Meredith & King, 2005), the same warming not seen in other areas. Deep-reaching warming from climate and oceanographic changes (Böning *et al.*, 2008, Gille, 2008), shifting ACC fronts and strengthening winds contributing to warming (Gille, 2008, Hogg *et al.*, 2008, Meredith & Hogg, 2006). | Sea ice expected to decrease in extent (Bracegirdle *et al.*, 2008), volume (Arzel *et al.*, 2006) and annual duration (i.e. lengthen open water season) (Stammerjohn *et al.*, 2012). Total marginal ice zone particularly affected (Arrigo & Thomas, 2004, Arrigo *et al.*, 2008). Freshening of water (Rintoul, 2007); iron & nutrient release from continental ice (Boyd *et al.*, 2012, Lannuzel *et al.*, 2007). Increased ocean surface layer stratification and increase in light conditions (Clark *et al.*, 2013) may cause phytoplankton blooms (Smith & Nelson, 1986) and increased primary production at sea-ice edge during spring and summer sea-ice retreat (Peck *et al.*, 2010). Micronutrients may seed water column with ice algae (Lizotte, 2001), but decreased sea-ice duration may counteract effects. | CO_2_ enrichment from increased atmospheric CO_2_, shift to acidic conditions (Midorikawa *et al.*, 2012), faster corrosion in cold polar waters as CO_2_ is more soluble (McNeil & Matear, 2008, Moy *et al.*, 2009). | Stratospheric ozone depletion has led to enhanced ultraviolet-B radiation over Antarctic regions (Karentz, 1994, Marchant *et al.*, 1994). Shallower mixed layer will increase mean irradiance levels (Constable *et al.*, 2014). |
| Expected impact on krill | Increased metabolic rates, enhance krill productivity (Constable *et al.*, 2014). Negative response around Sth Georgia where water temperatures have already increased (Mackey *et al.*, 2012) – further warming could raise metabolic costs to unsustainable levels (Constable *et al.*, 2014, Hill *et al.*, 2013, Murphy *et al.*, 2007, Wiedenmann *et al.*, 2008). Modelled uniform 1°C temperature rise produces pole-ward shift for euphausiids (Mackey *et al.*, 2012). Wiedenmann *et al.* (2008) growth model predicts increasing individual size within a length and weight cohort with increasing temperature in Antarctic Peninsula region (60-70°S), decreasing individual size with increasing temperature in warmer Sth Georgia region (50-60°S). | Ice-associated primary production (including ice algal production) expected to decline in some areas (e.g. marginal ice), reducing food availability (Arrigo & Thomas, 2004, Arrigo *et al.*, 2008, Vancoppenolle *et al.*, 2013a). Predicted changes in sea-ice thickness (Arzel *et al.*, 2006) may reduce habitat suitability for juveniles and impact on the availability of ice algae as food for krill (Meiners *et al.*, 2012, Vancoppenolle *et al.*, 2013b). Incomplete molting can occur from reduced juvenile habitat duration (Daly, 2004). The latter the timing of annual sea ice formation, the lower the food availability to krill, and the lower the growth rates and predicted survival rates of juvenile krill in particular (Quetin & Ross, 2009). | Affects physiology and carbonate deposition by calcifying organisms such as krill (Bednarsek *et al.*, 2012, Byrne, 2011, Constable *et al.*, 2014, Ingels *et al.*, 2012, Kawaguchi *et al.*, 2013, Kawaguchi *et al.*, 2011, Moy *et al.*, 2009). Increases the energetic costs of calcification (Hofmann *et al.*, 2010, Shi *et al.*, 2010). | Krill genome may be highly susceptible to increasing UV-B compared to other Antarctic organisms, based on DNA base composition (Jarman *et al.*, 1999). A significant correlation between krill density and Antarctic ozone depletion in the Antarctic Peninsula region has been reported suggesting direct and/or indirect UV impacts on krill density (Mangel *et al.*, 2010, Naganobu *et al.*, 1999). |
| Observed impact from experiments | Max growth rate at higher temperatures (4.48C) twice that of 0.68C (Poleck & Denys, 1982). In Antarctic krill, an increase in water temperature of 1°C is associated with a decrease in the mean intermolt period IMP of approximately 3.1d (Buchholz, 2003). Krill IMP significantly lower at elevated temperature treatments (Brown *et al.*, 2010, Saba *et al.*, 2012). | Decreased sea-ice associated with smaller krill population (Loeb *et al.*, 1997, Nicol *et al.*, 2000). Strong relationship between krill abundance/recruitment & winter sea ice extent (Atkinson *et al.*, 2004). Reduced ice-algae production could deprive krill of sufficient food to support their energy requirements, and therefore retard krill gonadal development and spawning (Loeb *et al.*, 1997). Timing of seasonal ice advance/retreat, and resultant duration of winter ice, affects habitat quality for krill (Quetin *et al.*, 2007). Krill recruitment linked to CHL anomalies from previous year (Richerson *et al.*, 2016). | Research combining experimental results and ocean circulation modelling shows survival of krill embryos may be negatively affected by increasing ocean acidity (Kawaguchi *et al.*, 2013, Kawaguchi *et al.*, 2011), with almost total hatching failure at 2000 ppm CO_2_ (Kawaguchi *et al.*, 2011). Ingestion rates of krill 3.5 times higher than krill ingestion rates at ambient, present day CO_2_ concentrations (Saba *et al.*, 2012). Under the RCP 8.5 scenario most of krill habitat will suffer at least 20% lower hatching success by 2100, with reductions of up to 60–70% in the Weddell Sea (Sth Atlantic) = threshold of no recruitment at 2000 ppm CO_2_. | Adults particularly susceptible to DNA damage and mortality due to UVB (Jarman *et al.*, 1999, Newman *et al.*, 1999). Adult behavioural changes, avoiding UVB and UVA (Newman *et al.*, 2003). Shifts in metabolism consistent with increased physiological costs associated with regulating internal acid-base equilibria, creating additional stress that may hamper growth and reproduction (Saba *et al.*, 2012). |
| Growth rate (adult) | +/- | - (reduced food)  + (incr. primary prod at ice edge) | - | - |
| Evidence | (Constable *et al.*, 2014, Poleck & Denys, 1982) | (Arrigo & Thomas, 2004, Arrigo *et al.*, 2008, Meiners *et al.*, 2012, Vancoppenolle *et al.*, 2013b) | (Kawaguchi *et al.*, 2013, Kawaguchi *et al.*, 2011) | (Newman *et al.*, 1999) |
| Growth rate (juv) | ? | +/- | - | - |
| Evidence |  | (Daly, 2004, Loeb *et al.*, 1997) | (Kawaguchi *et al.*, 2013, Kawaguchi *et al.*, 2011) | (Ban *et al.*, 2007) |
| Intermoult period | - | - (sea ice thickness) | - | ? |
| Evidence | (Brown *et al.*, 2010, Buchholz, 2003, Saba *et al.*, 2012) | (Daly, 2004) | (Bednarsek *et al.*, 2012, Byrne, 2011, Ingels *et al.*, 2012, Kawaguchi *et al.*, 2013, Kawaguchi *et al.*, 2011, Moy *et al.*, 2009) |  |
| Spawning/ recruitment | - | - | - | - |
| Evidence | (Loeb *et al.*, 1997) | (Loeb *et al.*, 1997, Siegel & Loeb, 1995) | (Byrne, 2011, Kurihara, 2008) | (Saba *et al.*, 2012) |
| Hatching success | ? | - | - | ? |
| Evidence | (Yoshida *et al.*, 2004) | (Daly, 2004, Loeb *et al.*, 1997) | (Kawaguchi *et al.*, 2011, Kurihara, 2008) |  |
| Larvae survival | ? | - | - | - |
| Evidence | (Ross *et al.*, 1988) | (Frazer *et al.*, 2002, Loeb *et al.*, 1997, Yoshida *et al.*, 2004) | (Kawaguchi *et al.*, 2013, Kawaguchi *et al.*, 2011) | (Ban *et al.*, 2007) |
| Juvenile survival | - | - | - | - |
| Evidence | (Saba *et al.*, 2012) | (Daly, 2004, Frazer *et al.*, 2002, Loeb *et al.*, 1997, Yoshida *et al.*, 2004) | (Kawaguchi *et al.*, 2011, Saba *et al.*, 2012) | (Ban *et al.*, 2007) |
| Adult survival | Location-dependent | + | - | - |
| Evidence | (Constable *et al.*, 2014) | ? | (Wynn-Edwards *et al.*, 2014, Wynn-Edwards, 2014) | (Newman *et al.*, 2003) |
| Food availability/ feeding success | + | + (during melt), - overall, esp. in marginal ice zone | - | - |
| Evidence | (Constable *et al.*, 2014) | (Frazer *et al.*, 2002, Loeb *et al.*, 1997, Massom & Stammerjohn, 2010, Yoshida *et al.*, 2004) | (Wynn-Edwards *et al.*, 2014, Wynn-Edwards, 2014) | (Saba *et al.*, 2012) |

**Supplementary references**

Arrigo KR, Thomas DN (2004) Large scale importance of sea ice biology in the Southern Ocean. Antarctic Science*,* **16**, 471-486.

Arrigo KR, Van Dijken G, Pabi S (2008) Impact of a shrinking Arctic ice cover on marine primary production. Geophysical Research Letters*,* **35**.

Arzel O, Fichefet T, Goosse H (2006) Sea ice evolution over the 20th and 21st centuries as simulated by current AOGCMs. Ocean Modelling*,* **12**, 401-415.

Atkinson A, Siegel V, Pakhomov E, Rothery P (2004) Long-term decline in krill stock and increase in salps within the Southern Ocean. Nature*,* **432**, 100-103.

Ban S, Ohi N, Leong SCY, Takahashi KT, Riser CW, Taguchi S (2007) Effect of solar ultraviolet radiation on survival of krill larvae and copepods in Antarctic Ocean. Polar Biology*,* **30**, 1295-1302.

Bednarsek N, Tarling GA, Bakker DCE *et al.* (2012) Extensive dissolution of live pteropods in the Southern Ocean. Nature Geoscience*,* **5**, 881-885.

Böning CW, Dispert A, Visbeck M, Rintoul S, Schwarzkopf FU (2008) The response of the Antarctic Circumpolar Current to recent climate change. Nature Geoscience*,* **1**, 864-869.

Boyd PW, Arrigo K, Strzepek R, Dijken G (2012) Mapping phytoplankton iron utilization: Insights into Southern Ocean supply mechanisms. Journal of Geophysical Research: Oceans*,* **117**.

Bracegirdle TJ, Connolley WM, Turner J (2008) Antarctic climate change over the twenty first century. Journal of Geophysical Research: Atmospheres*,* **113**.

Brown M, Kawaguchi S, Candy S, Virtue P (2010) Temperature effects on the growth and maturation of Antarctic krill (Euphausia superba). Deep Sea Research Part II: Topical Studies in Oceanography*,* **57**, 672-682.

Buchholz F (2003) Experiments on the physiology of southern and northern krill, Euphausia superba and Meganyctiphanes norvegica, with emphasis on moult and growth–a review. Mar. Fresh. Behav. Physiol.*,* **36**, 229-247.

Byrne M (2011) Impact of ocean warming and ocean acidification on marine invertebrate life history stages: vulnerabilities and potential for persistence in a changing ocean. Oceanography and Marine Biology*,* **49**, 1–42.

Clark GF, Stark JS, Johnston EL, Runcie JW, Goldsworthy PM, Raymond B, Riddle MJ (2013) Light‐driven tipping points in polar ecosystems. Global Change Biology*,* **19**, 3749-3761.

Constable AJ, Melbourne-Thomas J, Corney SP *et al.* (2014) Climate change and Southern Ocean ecosystems I: how changes in physical habitats directly affect marine biota. Global Change Biology*,* **20**, 3004-3025.

Daly KL (2004) Overwintering growth and development of larval Euphausia superba: an interannual comparison under varying environmental conditions west of the Antarctic Peninsula. Deep-Sea Research Part Ii-Topical Studies in Oceanography*,* **51**, 2139-2168.

Frazer TK, Quetin LB, Ross RM (2002) Abundance, sizes and developmental stages of larval krill, Euphausia superba, during winter in ice-covered seas west of the Antarctic Peninsula. Journal of Plankton Research*,* **24**, 1067-1077.

Gille ST (2008) Decadal-scale temperature trends in the Southern Hemisphere ocean. Journal of Climate*,* **21**, 4749-4765.

Hill SL, Phillips T, Atkinson A (2013) Potential climate change effects on the habitat of Antarctic krill in the Weddell quadrant of the Southern Ocean. Plos One*,* **8**, e72246.

Hofmann GE, Barry JP, Edmunds PJ, Gates RD, Hutchins DA, Klinger T, Sewell MA (2010) The effect of ocean acidification on calcifying organisms in marine ecosystems: an organism-to-ecosystem perspective. Annual Review of Ecology, Evolution and Systematics*,* **41**, 127-147.

Hogg AMC, Meredith MP, Blundell JR, Wilson C (2008) Eddy heat flux in the Southern Ocean: Response to variable wind forcing. Journal of Climate*,* **21**, 608-620.

Ingels J, Vanreusel A, Brandt A *et al.* (2012) Possible effects of global environmental changes on Antarctic benthos: a synthesis across five major taxa. Ecology and Evolution*,* **2**, 453-485.

Jarman S, Elliott N, Nicol S, Mcminn A, Newman S (1999) The base composition of the krill genome and its potential susceptibility to damage by UV-B. Antarctic Science*,* **11**, 23-26.

Karentz D (1994) *Ultraviolet tolerance mechanisms in Antarctic marine organisms*, Wiley Online Library.

Kawaguchi S, Ishida A, King R *et al.* (2013) Risk maps for Antarctic krill under projected Southern Ocean acidification. Nature Climate Change*,* **3**.

Kawaguchi S, Kurihara H, King R *et al.* (2011) Will krill fare well under Southern Ocean acidification? Biology Letters*,* **7**, 288-291.

Kurihara H (2008) Effects of CO2-driven ocean acidification on the early developmental stages of invertebrates. Marine Ecology Progress Series*,* **373**, 275-284.

Lannuzel D, Schoemann V, De Jong J, Tison J-L, Chou L (2007) Distribution and biogeochemical behaviour of iron in the East Antarctic sea ice. Marine Chemistry*,* **106**, 18-32.

Lizotte MP (2001) The contributions of sea ice algae to Antarctic marine primary production. American Zoologist*,* **41**, 57-73.

Loeb V, Siegel V, Holmhansen O, Hewitt R, Fraser W, Trivelpiece W, Trivelpiece S (1997) Effects of sea-ice extent and krill or salp dominance on the Antarctic food web. Nature*,* **387**, 897-900.

Mackey A, Atkinson A, Hill S, Ward P, Cunningham N, Johnston N, Murphy E (2012) Antarctic macrozooplankton of the southwest Atlantic sector and Bellingshausen Sea: Baseline historical distributions (Discovery Investigations, 1928–1935) related to temperature and food, with projections for subsequent ocean warming. Deep Sea Research Part II: Topical Studies in Oceanography*,* **59**, 130-146.

Mangel M, Richerson K, Cresswell KA, Wiedenmann JR (2010) Modelling the effects of UV radiation on the survival of Antarctic krill (Euphausia superba Dana) in the face of limited data. Ecological Modelling*,* **221**, 2095-2101.

Marchant DR, Denton GH, Bockheim JG, Wilson SC, Kerr AR (1994) Quaternary changes in level of the upper Taylor Glacier, Antarctica: implications for paleoclimate and East Antarctic Ice Sheet dynamics. Boreas*,* **23**, 29-43.

Massom RA, Stammerjohn SE (2010) Antarctic sea ice change and variability–physical and ecological implications. Polar Science*,* **4**, 149-186.

Mcneil BI, Matear RJ (2008) Southern Ocean acidification: A tipping point at 450-ppm atmospheric CO2. Proceedings of the National Academy of Sciences*,* **105**, 18860-18864.

Meiners K, Vancoppenolle M, Thanassekos S *et al.* (2012) Chlorophyll a in Antarctic sea ice from historical ice core data. Geophysical Research Letters*,* **39**.

Meredith MP, Hogg AM (2006) Circumpolar response of Southern Ocean eddy activity to a change in the Southern Annular Mode. Geophysical Research Letters*,* **33**.

Meredith MP, King JC (2005) Rapid climate change in the ocean west of the Antarctic Peninsula during the second half of the 20th century. Geophysical Research Letters*,* **32**.

Midorikawa T, Inoue HY, Ishii M *et al.* (2012) Decreasing pH trend estimated from 35-year time series of carbonate parameters in the Pacific sector of the Southern Ocean in summer. Deep Sea Research Part I: Oceanographic Research Papers*,* **61**, 131-139.

Moy CM, Moreno PI, Dunbar RB, Kaplan MR, Francois J-P, Villalba R, Haberzettl T (2009) Climate change in southern South America during the last two millennia. In: *Past climate variability in South America and surrounding regions.*  pp Page., Springer.

Murphy EJ, Trathan PN, Watkins JL *et al.* (2007) Climatically driven fluctuations in Southern Ocean ecosystems. Proceedings of the Royal Society of London B: Biological Sciences*,* **274**, 3057-3067.

Naganobu M, Kutsuwada K, Sasai Y, Taguchi S, Siegel V (1999) Relationships between Antarctic krill (Euphausia superba) variability and westerly fluctuations and ozone depletion in the Antarctic Peninsula area. Journal of Geophysical Research: Oceans*,* **104**, 20651-20665.

Newman SJ, Nicol S, Ritz D, Marchant H (1999) Susceptibility of Antarctic krill (Euphausia superba Dana) to ultraviolet radiation. Polar Biology*,* **22**, 50-55.

Newman SJ, Ritz D, Nicol S (2003) Behavioural reactions of Antarctic krill (Euphausia superba Dana) to ultraviolet and photosynthetically active radiation. Journal of Experimental Marine Biology and Ecology*,* **297**, 203-217.

Nicol S, Pauly T, Bindoff NL *et al.* (2000) Ocean circulation off east Antarctica affects ecosystem structure and sea-ice extent. Nature*,* **406**, 504-507.

Peck LS, Barnes DK, Cook AJ, Fleming AH, Clarke A (2010) Negative feedback in the cold: ice retreat produces new carbon sinks in Antarctica. Global Change Biology*,* **16**, 2614-2623.

Perrin WF, Wursig B (2009) *Encyclopedia of marine mammals,* San Diego, Academic Press.

Poleck T, Denys C (1982) Effect of temperature on the molting, growth and maturation of the Antarctic krill Euphausia superba (Crustacea: Euphausiacea) under laboratory conditions. Marine Biology*,* **70**, 255-265.

Quetin LB, Ross RM (2009) *Life under Antarctic Pack Ice: A Krill Perspective*.

Quetin LB, Ross RM, Fritsen CH, Vernet M (2007) Ecological responses of Antarctic krill to environmental variability: can we predict the future? Antarctic Science*,* **19**, 253-266.

Ratnarajah L, Melbourne-Thomas J, Marzloff MP *et al.* (2016) A preliminary model of iron fertilisation by baleen whales and Antarctic krill in the Southern Ocean: Sensitivity of primary productivity estimates to parameter uncertainty. Ecological Modelling*,* **320**, 203-212.

Richerson K, Santora JA, Mangel M (2016) Climate variability and multi-scale assessment of the krill preyscape near the north Antarctic Peninsula. Polar Biology, 1-15.

Rintoul SR (2007) Rapid freshening of Antarctic Bottom Water formed in the Indian and Pacific oceans. Geophysical Research Letters*,* **34**.

Ross R, Quetin L, Kirsch E (1988) Effect of temperature on developmental times and survival of early larval stages of Euphausia superba Dana. Journal of Experimental Marine Biology and Ecology*,* **121**, 55-71.

Saba GK, Schofield O, Torres JJ, Ombres EH, Steinberg DK (2012) Increased feeding and nutrient excretion of adult Antarctic krill, Euphausia superba, exposed to enhanced carbon dioxide (CO 2).

Shi D, Xu Y, Hopkinson BM, Morel FM (2010) Effect of ocean acidification on iron availability to marine phytoplankton. Science*,* **327**, 676-679.

Siegel V, Loeb V (1995) Recruitment of Antarctic krill Euphausia superba and possible causes for its variability. Marine Ecology Progress Series*,* **123**, 45-56.

Smith WO, Nelson DM (1986) Importance of ice edge phytoplankton production in the Southern Ocean. Bioscience*,* **36**, 251-257.

Stammerjohn S, Massom RA, Rind D, Martinson D (2012) Regions of rapid sea ice change: An inter-hemispheric seasonal comparison. Geophysical Research Letters*,* **39**.

Tamura T, Ichii T, Fujise Y (1997) Consumption of krill by minke whales in Areas IV and V of the Antarctic. IWC Scientific Committee working paper SC/M97/17, 9p. (unpublished). pp Page.

Tamura T, Konishi K (2006) Food habit and prey consumption of Antarctic minke whale Balaenoptera bonaerensis in JARPA research area. The Institute of Cetacean Research, Tokyo.

Tamura T, Konishi K (2009) Feeding habits and prey consumption of Antarctic minke whale (Balaenoptera bonaerensis) in the Southern Ocean. Journal of Northwest Atlantic fishery science*,* **42**, 13-25.

Tulloch VJD, Plagányi ÉE, Matear R, Brown CJ, Richardson AJ (2017) Ecosystem modelling to quantify the impact of historical whaling on Southern Hemisphere baleen whales. Fish and Fisheries*,* **doi: 10.1111/faf.12241**.

Vancoppenolle M, Bopp L, Madec G, Dunne J, Ilyina T, Halloran PR, Steiner N (2013a) Future Arctic Ocean primary productivity from CMIP5 simulations: Uncertain outcome, but consistent mechanisms. Global Biogeochemical Cycles*,* **27**, 605-619.

Vancoppenolle M, Meiners KM, Michel C *et al.* (2013b) Role of sea ice in global biogeochemical cycles: emerging views and challenges. Quaternary science reviews*,* **79**, 207-230.

Wiedenmann J, Cresswell K, Mangel M (2008) Temperature-dependent growth of Antarctic krill: predictions for a changing climate from a cohort model. Marine Ecology Progress Series*,* **358**, 191.

Wynn-Edwards C, King R, Davidson A *et al.* (2014) Species-specific variations in the nutritional quality of Southern Ocean phytoplankton in response to elevated pCO2. Water*,* **6**, 1840-1859.

Wynn-Edwards CA (2014) Effects of ocean acidification on the nutritional quality of Antarctic phytoplankton as food for Euphausia suberda larvae. University of Tasmania.

Yoshida T, Toda T, Hirano Y, Matsuda T, Kawaguchi S (2004) Effect of temperature on embryo development time and hatching success of the Antarctic krill Euphausia superba Dana in the laboratory. Marine and Freshwater Behaviour and Physiology*,* **37**, 137-145.
